# The mRNP remodeling mediated by UPF1 promotes rapid degradation of replication-dependent histone mRNA

**DOI:** 10.1093/nar/gku610

**Published:** 2014-07-12

**Authors:** Junho Choe, Sang Ho Ahn, Yoon Ki Kim

**Affiliations:** Division of Life Sciences, Korea University, Seoul 136–701, Republic of Korea

## Abstract

Histone biogenesis is tightly controlled at multiple steps to maintain the balance between the amounts of DNA and histone protein during the cell cycle. In particular, translation and degradation of replication-dependent histone mRNAs are coordinately regulated. However, the underlying molecular mechanisms remain elusive. Here, we investigate remodeling of stem-loop binding protein (SLBP)-containing histone mRNPs occurring during the switch from the actively translating mode to the degradation mode. The interaction between a CBP80/20-dependent translation initiation factor (CTIF) and SLBP, which is important for efficient histone mRNA translation, is disrupted upon the inhibition of DNA replication or at the end of S phase. This disruption is mediated by competition between CTIF and UPF1 for SLBP binding. Further characterizations reveal hyperphosphorylation of UPF1 by activated ATR and DNA-dependent protein kinase upon the inhibition of DNA replication interacts with SLBP more strongly, promoting the release of CTIF and eIF3 from SLBP-containing histone mRNP. In addition, hyperphosphorylated UPF1 recruits PNRC2 and SMG5, triggering decapping followed by 5′-to-3′ degradation of histone mRNAs. The collective observations suggest that both inhibition of translation and recruitment of mRNA degradation machinery during histone mRNA degradation are tightly coupled and coordinately regulated by UPF1 phosphorylation.

## INTRODUCTION

In mammals, two different types of cap-binding proteins initiate translation in the cytoplasm (1). One is a nuclear cap-binding complex (CBC), a heterodimer of nuclear cap-binding proteins 80 and 20 (CBP80/20), and the other is a cytoplasmic cap-binding protein, eukaryotic translation initiation factor (eIF) 4E. In the nucleus, newly synthesized mRNAs first associate with CBC and are exported from the nucleus to cytoplasm via the nuclear pore complex. During or after mRNA export, the CBC is replaced by eIF4E in the cytoplasm [i.e. CBC-bound mRNAs are precursors of eIF4E-bound mRNAs (1,2)].

Although both CBC and eIF4E have a common ability to recruit ribosomes, the mechanisms are quite different. In the case of translation of CBC-bound mRNAs, the cap-bound CBC recruits CBC-dependent translation initiation factor (CTIF), which is an eIF4G-like protein, by direct interaction (3). Since CTIF is enriched in the cytoplasmic side of the nuclear envelope and is also localized to the nucleus (3), the recruitment of CTIF toward the 5′-end of mRNA by CBC may occur in the nucleus or during mRNA export via the nuclear pore complex. Our recent data showed that CTIF directly binds to eIF3g (4), which is a component of eIF3 complex. This observation suggests that CBP80-CTIF at the 5′-end of mRNA recruits the eIF3 complex that, in turn, recruits the small subunit of ribosome (40S), initiating the first round (or pioneer round) of translation (1,3,5). However, our and other groups have reported that the translation of CBC-bound mRNAs occurs multiple times (3,6–8). Therefore, we will hereafter refer to this as CBC-dependent translation (CT), regardless of the number of translation initiations (3).

The cap structure-bound CBC is replaced by eIF4E with the action of importin α/β in a translation-independent manner (9). Then, the cap-bound eIF4E recruits eIF4GI/II, which recruits eIF3 and eventually the 40S ribosome, driving the expression of bulk amount of polypeptides in the cytoplasm (10). For comparison, we hereafter refer to this as eIF4E-dependent translation (ET).

Besides molecular differences in CT and ET, these two modes of translation play distinct roles in gene expression. For instance, it is known that nonsense-mediated mRNA decay (NMD) is mainly coupled to the first round of translation (5,11,12). The NMD is a well-characterized mRNA surveillance mechanism by which aberrant transcripts or cellular transcripts harboring premature translation termination codons are selectively recognized and eliminated by an action of UPF1 during translation termination (13–17). Since CBC-bound mRNAs are precursors of eIF4E-bound mRNAs (1,2), it is expected that the first round of translation is largely driven by CBC rather than eIF4E. In addition to NMD, the translation of a majority of replication-dependent histone mRNAs in the cytoplasm largely occurs on CBC-bound mRNAs rather than eIF4E-bound mRNAs (18). Accordingly, degradation of replication-dependent histone mRNAs largely occurs on CBC-bound mRNAs (18).

The abundance of replication-dependent histone mRNA (hereafter called histone mRNA, if not specified) is tightly controlled by a series of mechanisms (19,20). Tight regulation of histone mRNA translation and stability are essential for balancing the amounts of DNA and histone protein during cell cycle, which is achieved by a unique stem-loop structure at the 3′-end of histone mRNAs, instead of conventional polyadenylated sequences found in most eukaryotic mRNAs. The unique stem-loop structure provides a binding platform for stem-loop binding protein (SLBP), which plays a critical role in the entire lifespan of histone mRNAs including pre-mRNA processing, mRNA export, translation and degradation (19–21). A recent study showed that overall shape rather than the sequence of the stem loop is important for SLBP binding, although a single nucleotide in the stem region is directly recognized by SLBP (22).

Here, we investigate possible remodeling of histone messenger ribonucleoprotein (mRNP) during the transition from active translation to rapid degradation. We show that ataxia telangiectasia and Rad3-related kinase (ATR) and DNA-dependent protein kinase (DNA-PK), both of which belong to the phosphoinositide 3-kinase-related kinases (PIKKs) family (23), are activated and trigger UPF1 phosphorylation upon the inhibition of DNA replication. The hyperphosphorylated UPF1 plays dual roles in remodeling of histone mRNP. It interacts with SLBP more strongly, promoting the release of CTIF and eIF3 from SLBP-containing histone mRNPs, consequently repressing translation of histone mRNAs. At the same time, it more strongly associates with proline-rich nuclear receptor coregulatory protein 2 (PNRC2) and SMG5, both of which are previously known to trigger NMD (24–30), consequently rendering histone mRNAs more vulnerable to degradation. In this way, SLBP-containing histone mRNPs undergo dramatic remodeling mediated by UPF1 phosphorylation during the switch from active translation mode to rapid degradation mode.

## MATERIALS AND METHODS

### Plasmid constructions

The following plasmids were used in this study: pcDNA3-FLAG and pcDNA3-FLAG-CTIF (3); pcDNA3-FLAG-SLBP-WT, pcDNA3-FLAG-SLBP-W75A, pCMV-Myc-SLBP, pCMV-Myc-SLBP(1–127), pCMV-Myc-SLBP(128–270), pBiFC-SLBP-VN, pBiFC-CTIF-VC and pRSF-GST-SLBP(1–127) (18); pcDNA3-FLAG-UPF1(WT), pcDNA3-FLAG-UPF1(G495R/G497E) (renamed pcDNA3-FLAG-UPF1-HP in this study), and pCMV-Myc-PNRC2 (25); pMS2-HA and pCMV-Myc-UPF1(WT) (31); pCMV-Myc-UPF1(HP) (6); His-CTIF(365–598) (4); pmCMV-Gl Norm or Ter (32); pmCMV-GPx1 Norm or Ter (33) and phCMV-MUP (34).

To construct plasmid pcDNA3-FLAG-SLIP1, which encodes FLAG tagged full-length human SLIP1 cDNA, a NotI/KpnI fragment from pcDNA3-FLAG was ligated to a polymerase chain reaction (PCR)-amplified fragment that contained human SLIP1 cDNA and was digested with NotI/KpnI, respectively. The full-length human SLIP1 cDNA was amplified using pCMV-SPORT6-SLIP1 (18) and two oligonucleotides: 5′-CGGAATTCCGGCGGCCGCTATGGGGGAGCCCAGTAGAGAGGAGTATAAAATC-3′ (sense) and 5′-GGGGTACCCTAGTCGGAGACTTCGCTGTAGTAATACTTGTG-3′ (antisense), where the underlined nucleotides specify the NotI and KpnI sites, respectively.

To construct RLuc-8bs plasmid, a BamHI/EcoRI fragment of no intron (35) was ligated to a BamHI/EcoRI fragment of PCR product that contained eight tandem repeats of MS2bs. The PCR was performed using pcFLuc-MS2bs (31) as a template and two oligonucleotides: 5′-CGGGATCCCCTTTGTTCCCTAAGTCCAACTACC-3′ (sense) and 5′-GGAATTCCAATGTATCTTATCATTCTGCTCG-3′ (antisense), where the underlined nucleotides specify the BamHI and EcoRI sites, respectively.

To generate RLuc-SL-8bs plasmid, a HindIII/BamHI fragment of RLuc-8bs plasmid was ligated to two fragments: (i) a HindIII/NheI fragment of RLuc-8bs plasmid, which contains *RLuc* cDNA and (ii) a NheI/BamHI fragment of a PCR product that contains cDNA corresponding to *HIST2H2AA* mRNA 3′UTR lacking U7 snRNP-binding site. The PCR was performed using (i) reverse-transcribed total-cell cDNAs from HeLa cells as a template and (ii) two oligonucleotides: 5′-CGGCTAGCGCTGACGTCCGGCCCAAGTGGGCC-3′ (sense) and 5′-CGGGATCCTGGGTGGCTCTGAAAAGAGCCTTT-3′ (antisense), where the underlined nucleotides specify the NheI and BamHI sites, respectively.

To generate pMS2-HA-MBP, the XhoI/NotI fragment of pMS2-HA-PNRC2 (25) was ligated to the XhoI/NotI fragment of the PCR product that was amplified using pMAL-p4X (New England Biolabs) as a template and two oligonucleotides: 5′-CCGCTCGAGATGAAAATAAAAACAGGTGCACGCATC-3′ (sense) and 5′-ATAAGAATGCGGCCGCTTAAGTCTGCGCGTCTTTCAGGGCTTCATC-3′ (antisense), where the underlined nucleotides specify the XhoI and NotI sites, respectively.

To construct pHA-MBP, the NheI/NotI fragment of pMS2-HA-MBP was ligated to the NheI/NotI fragment of the PCR product that was amplified using pMS2-HA-MBP as a template and two oligonucleotides: 5′-CTAGCTAGCCGCCATGTACCCATACGACGTTCCAGACTACGCTC-3′ (sense) and 5′-ATAAGAATGCGGCCGCTTAAGTCTGCGCGTCTTTCAGGGCTTCATC-3′ (antisense), where the underlined nucleotides specify the NheI and NotI sites, respectively.

For bacterial production of His-UPF1(295–914), a BamHI/XhoI fragment of pGEX-6p-1-UPF1(295–914) (a gift from Haiwei Song) was cloned into a BamHI/XhoI fragment of FP-His vector, a modified pET vector (Novagen) containing a cleavable TEV protease site at the amino-terminus.

### Cell culture and transfections

Cells used in this study were grown in Dulbecco's Modified Eagle's Medium (DMEM; Lonza) containing 10% fetal bovine serum (FBS; Lonza). For immunoprecipitation (IP), HeLa and HEK293T cells were transiently transfected with the indicated plasmids using either calcium-phosphate or Lipofectamin 2000 (Invitrogen), as previously described (3,25,30,36). For siRNA transfection, HeLa cells were transfected with 100 nM *in vitro*-synthesized siRNA (Invitrogen) using Oligofectamine (Invitrogen), as previously described (3,25,30,36). The following siRNAs were used: Control siRNA (31), *UPF1* siRNA (31), *CTIF* siRNA (3), *PNRC2* siRNA (25), *SMG5* siRNA (24), *SMG6* siRNA (27,28), *SMG7* siRNA (24), TUTase 1 siRNA (37) and TUTase 3 siRNA (37).

When indicated, cells were treated with 5 mM hydroxyurea (HU; Sigma-Aldrich) for 20 min or 60 min before cell harvest. When indicated, cells were treated with ATM/ATR inhibitor (20 μM; Calbiochem), ATM inhibitor (1.3 μM; Calbiochem), LY294002 (140 μM; Calbiochem) or caffeine (15 mM; Sigma) for 2 h before cell harvest, or okadaic acid (OA; 75 nM; Sigma-Aldrich) for 5 h before cell harvest.

### Quantitative real-time PCR and RT-PCR using α-[^32^P]-dATP

Quantitative real-time PCR (qRT-PCR) analyses using the LightCycler system (Roche) were performed with single-stranded complementary DNA and gene-specific oligonucleotides with the LightCycler 480 SYBR Green I Master Mix (Roche) as previously described (3,25,30). The RT-PCR using α-[^32^P]-dATP was performed as previously described (3,25,30). Oligonucleotides used for amplification of *HIST2H2AA* mRNAs, *HIST1H1C* mRNAs, *H3F3A* mRNAs, *Gl* mRNAs, *GPx1* mRNAs, *MUP* mRNAs, RLuc-8bs mRNAs, RLuc-SL-8bs mRNAs, β-actin mRNAs and *GAPDH* mRNAs were previously reported (3,18).

TUTase 1 mRNAs and TUTase 3 mRNAS were amplified using two specific oligonucleotides: 5′-TCGTGAGGTTCTCACACCAG-3′ (sense) and 5′-GATGACCATCATTGTAAGGG-3′ (antisense) for TUTase 1 mRNAs, and 5′-GATGCTTGCATCCCCAATAC-3′ (sense) and 5′-TTGGTGATACAGCATGACTC-3′ (antisense) for TUTase 3 mRNAs.

For amplification of uridylated *HIST2H2AA* mRNAs and uridylated *HIST1H1C* mRNAs, oligo d(A)_18_ were used to synthesize cDNA. qRT-PCRs were performed using the same oligonucleotides used for amplification of *HIST2H2AA* mRNAs and *HIST1H1C* mRNAs, respectively.

### IP and western blotting

Two days after plasmid transfection, cells were harvested and centrifuged, suspended in 500 μl of NET-2 buffer [50 mM Tris-HCl (pH 7.4), 150 mM NaCl, 1 mM phenylmethanesulfonylfluoride, 2 mM benzamidine, 0.05% NP-40] containing 100 U of RNase inhibitor (Fermentas) and sonicated using a Branson Sonifier 250. After centrifugation, the supernatant was subjected to IP as previously described (3,25,30). Where indicated, the supernatant was treated with 2 μg of RNase A (Sigma) before IP and incubated for 15 min at 37°C. When indicated, cells were incubated with 1% of formaldehyde (Sigma-Aldrich) in phosphate-buffered saline for 10 min before harvesting (38).

The following antibodies were used for western blotting: FLAG and β-actin (Sigma-Aldrich), Myc (Calbiochem), HA (Roche), CBP80 (4), eIF4E, eIF3a, and phospho-S/TQ (Cell Signaling Technologies), eIF3b and eIF3c (Santa Cruz Biotechnology), eIF4GI (a gift from S. K. Jang), CTIF (3), SLBP (39), SLIP1 (40), UPF1 (a gift from L. E. Maquat), PNRC2 and DCP1A (25), SMG5 (Abcam), SMG6 (a gift from S. Ohno), SMG7 (Bethyl), GST (Amersham) and 6xHis (GE Healthcare).

### Bimolecular fluorescence complementation assay

Bimolecular fluorescence complementation (BiFC) assay using HeLa cells was performed as previously described (18), except that HeLa cells were co-transfected with the plasmids pBiFC-SLBP-VN and pBiFC-CTIF-VC and were immediately treated with 5 mM HU in DMEM containing 10% FBS for 8 h. The cells were observed with a LSM510 META confocal microscope (Carl Zeiss). Venus enhanced fluorescent protein (VFP) expression was measured using excitation at 500 nm and emission at 535 nm (41).

### Double thymidine block

For the synchronization and release of cell cycle, the double thymidine block (DTB) method was performed as previously described (18).

### Protein expression, purification and GST pull-down assay

GST-SLBP(1–127) was overexpressed in *Escherichia coli* BL21(DE3) by the addition of 1 mM isopropylthio-β-galactoside when the optical density (600 nm value) was 0.5. The cells were incubated for additional 20 h at 18°C. Total-cell extracts were directly used for *in vitro* GST pull-down experiments without further purification (18). Recombinant His-CTIF(365–598) and His-UPF1(295–914) were purified as previously described (4).

## RESULTS

### CTIF–SLBP interaction is abolished upon the inhibition of DNA replication or at the end of S phase

Previous reports showed that rapid degradation of histone mRNAs requires translation promoted by SLBP (42–44) and that SLBP is also important for rapid degradation of histone mRNA upon the inhibition of DNA replication or at the end of S phase during cell cycle (19,20). Recently, our group identified CTIF as SLBP-interacting protein and found that the CTIF–SLBP interaction is important for efficient histone mRNA translation (18). Furthermore, we showed that rapid degradation of histone mRNA largely takes place during CT but not ET (18). These previous observations led us to test the histone mRNP remodeling that takes place during the transition from active translation to rapid degradation.

**Figure 1.**
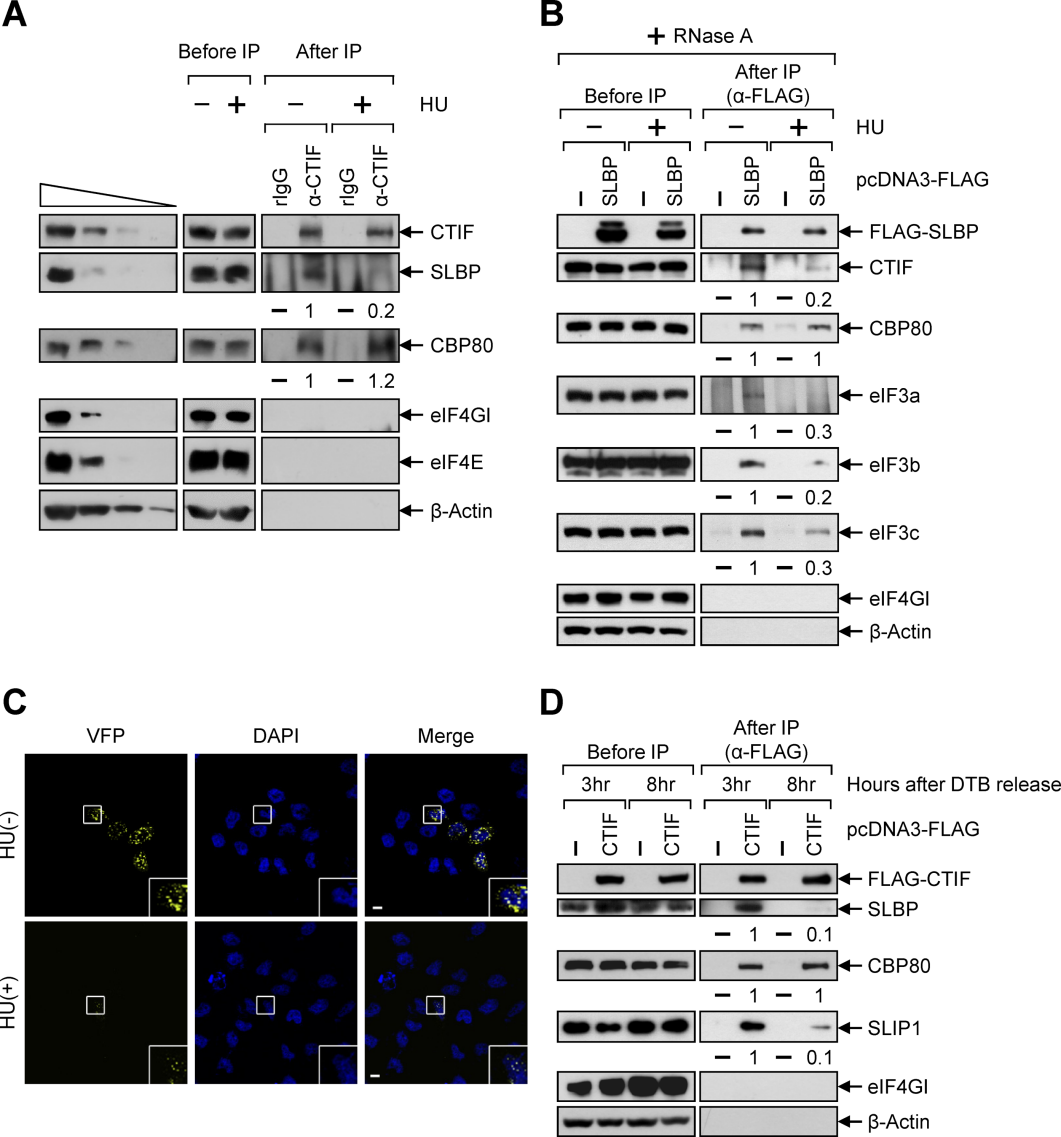
CTIF–SLBP interaction is disrupted upon the inhibition of DNA replication or at the end of S phase. (**A**) IPs of FLAG-CTIF. HEK293T cells were either untreated or treated with 5 mM HU for 20 min before cell harvest. Total-cell extracts were subjected to IP using either α-CTIF antibody or nonspecific rabbit (r) IgG. The levels of co-immunopurified endogenous SLBP and CBP80 were normalized to the levels of immunopurified endogenous CTIF. The normalized levels obtained in the IP of CTIF without the treatment of HU were arbitrarily set to 1. (**B**) IPs of FLAG-SLBP. As in Figure 1A, except that cells were transiently transfected with plasmid expressing either FLAG or FLAG-SLBP. (**C**) BiFC analysis for CTIF–SLBP interaction upon HU treatment. HeLa cells were transiently co-transfected with plasmids expressing SLBP-VN and CTIF-VC. Right after transfection, cells were either untreated (-) or treated (+) with 5 mM HU. After 8 h, the cells were fixed and observed by confocal microscopy. (**D**) IPs of FLAG-CTIF at the different stage of cell cycle. HEK293T cells transiently expressing FLAG-CTIF were synchronized by DTB. The extracts obtained from cells harvested 3 h (mid-S phase) and 8 h (late S phase) after the release of DTB were subjected to IPs using α-FLAG-conjugated agarose beads. The levels of co-immunopurified cellular SLBP, CBP80 and SLIP1 were normalized to the levels of immunopurified FLAG-CTIF. The normalized levels obtained in the IP of FLAG-CTIF at mid-S phase were arbitrarily set to 1. Each panel of results is representative of at least three independently performed transfections followed by IPs or BiFC analyses.

The results of IPs revealed that the amount of endogenous SLBP that co-immunopurified with endogenous CTIF was reduced 5-fold following treatment with HU for 20 min (Figure 1A), suggesting that the inhibition of DNA replication promotes the dissociation of CTIF and SLBP. Of note, the association between CBP80 and CTIF was not affected, which is reasonable because many cellular mRNAs are considered to use CTIF for CT (3). Consistent with previous reports showing that CT preferentially uses CTIF rather than eIF4GI/II (3,4,18), eIF4GI was not observed in the IP of CTIF under our conditions.

The reciprocal IP using FLAG-SLBP showed that the amounts of endogenous CTIF, eIF3a, eIF3b and eIF3c co-immunopurified with FLAG-SLBP were reduced by 5-, 3-, 5- and 3-fold, respectively, upon HU treatment (Figure 1B). There was no significant change in the association between CBP80 and SLBP upon HU treatment, consistent with the previous report showing the direct interaction between CBP80 and SLBP (45). Furthermore, an introduction of the W75A mutation in the translation-activating domain of SLBP caused the dissociation of CTIF and eIF3 components from SLBP-containing complex (Supplementary Figure S1). It is known that the corresponding mutant fails to interact with CTIF (18) and loses the ability to enhance histone mRNA translation without affecting histone pre-mRNA processing (40,42,46). Given that a CTIF directly interacts with eIF3 (4), these results suggest that the presence of eIF3 in SLBP-containing complex is due to the CTIF–SLBP interaction.

To more clearly demonstrate that the CTIF–SLBP interaction is disrupted upon HU treatment, we employed BiFC approaches (41) using SLBP-fused N-terminal fragment of yellow Venus enhanced fluorescent protein (SLBP-VN) and CTIF-fused C-terminal fragment of yellow Venus enhanced fluorescent protein (CTIF-VC). The results showed the significant enrichment of fluorescent signals on the cytoplasmic side of the perinuclear region when cells expressed both SLBP-VN and CTIF-VC (Figure 1C), indicating the *in vivo* interaction between CTIF and SLBP. The fluorescent signals significantly disappeared upon HU treatment (Figure 1C). We could not observe any significant fluorescent signals when SLBP(W75A)-VN was coexpressed with CTIF-VC under the same conditions (18). All these results suggest that the inhibition of DNA replication promotes the release of CTIF from SLBP-containing complex.

It is known that SLIP1 forms heterodimer or a heterotetramer with SLBP (47) and is required for histone mRNA translation (40,47). Therefore, we next investigated SLBP-interacting protein 1 (SLIP1)-containing complex using IP. The results showed that SLBP and CTIF, but not eIF4E and eIF4GI, co-immunopurified with FLAG-SLIP1 in an RNase A-resistant manner (Supplementary Figure S2A), suggesting the association between CTIF and SLIP1. In addition, whereas comparable amounts of co-immunopurified SLBP were observed in the IP of FLAG-SLIP1, less CTIF co-immunopurified with FLAG-SLIP1 upon treatment with HU (Supplementary Figure S2B), suggesting that HU treatment promotes CTIF-SLIP1 dissociation without affecting SLIP1–SLBP interaction.

The release of CTIF from SLBP-containing complex was further demonstrated during the cell cycle (Figure 1D). The cell cycles of HEK293T cells transiently expressing FLAG-CTIF were synchronized and released by DTB. The extracts obtained from cells harvested at 3 h (mid-S phase) and 8 h (late S phase) after the release of DTB were subjected to IPs using α-FLAG-conjugated agarose beads. The results showed that SLBP and SLIP1 efficiently co-immunopurified with FLAG-CTIF at mid-S phase. On the other hand, ∼10-fold less SLBP and SLIP1 co-immunopurified with FLAG-CTIF at the late S phase. Consistent with the results in Figure 1A, CBP80-CTIF association was not significantly changed during cell cycle. All results in Figure 1 suggest that CTIF is released from the SLBP-containing histone mRNPs upon the inhibition of DNA replication or at the end of S phase.

### Artificial insertion of histone stem-loop structure into 3′UTR renders mRNA more associated with CBC and CTIF, the latter of which is released from mRNP upon HU treatment

**Figure 2.**
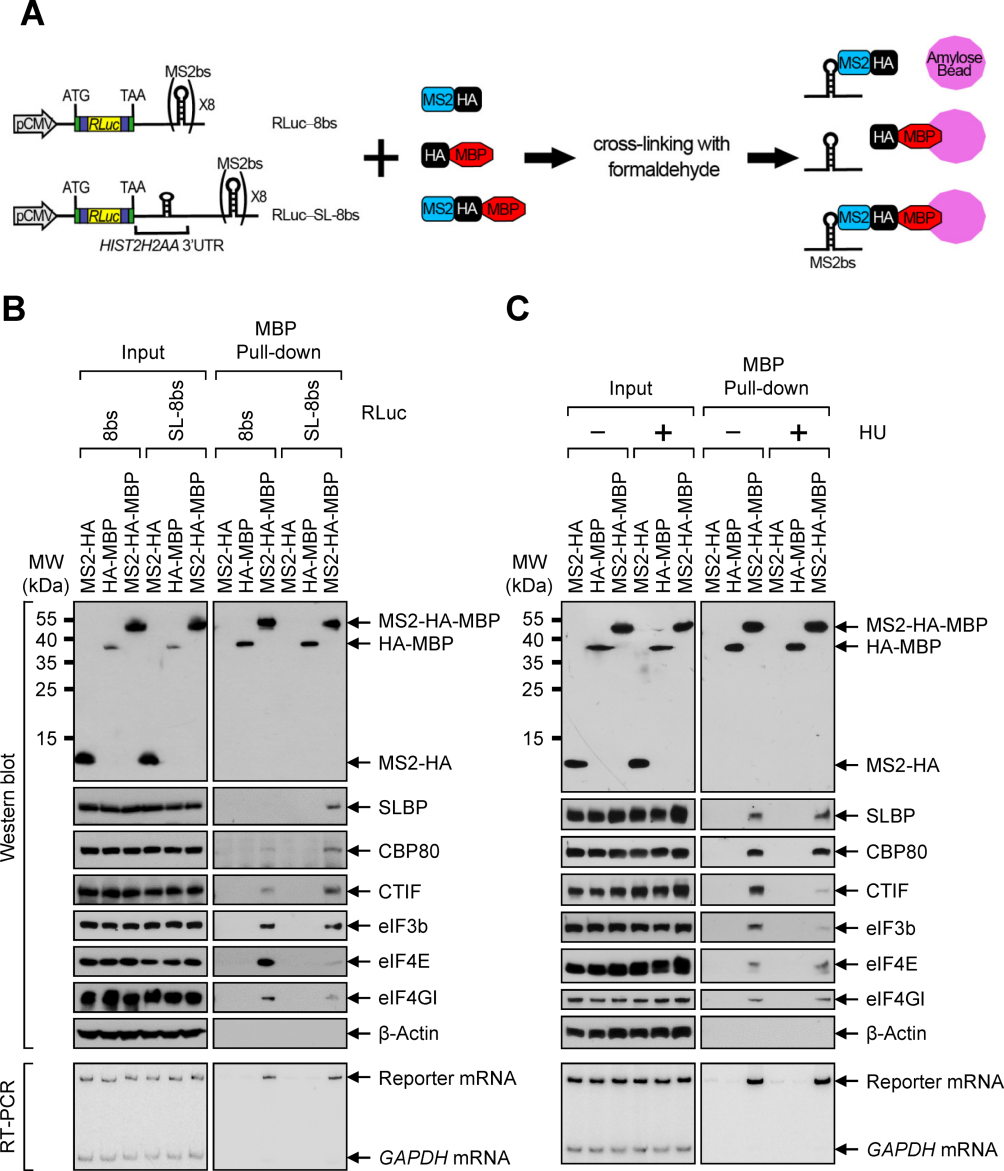
Artificial insertion of histone stem-loop structure to the 3′UTR of the reporter mRNA promotes an associate of CBC and CTIF with mRNA in a HU-independent and -dependent manner, respectively. (**A**) A schematic diagram of the MBP pull-down assay. HEK293T cells were co-transfected with a reporter plasmid either RLuc-8bs or RLuc-SL-8bs, and an effector plasmid expressing MS2-HA, HA-tagged MBP (HA-MBP) or MS2-HA-MBP. Two days after the transfection, the cells were cross-linked with formaldehyde. Total-cell extracts were harvested and subjected to MBP pull-down assay using amylose beads. (**B**) MBP pull-down assay using RLuc-8bs mRNA and RLuc-SL-8bs mRNA. The proteins and the reporter mRNAs purified by the MBP pull-down assay were analyzed by western blotting using the indicated proteins (top) and RT-PCR using specific oligonucleotides and α-[^32^P]-dATP, respectively. (**C**) MBP pull-down assay using RLuc-SL-8bs mRNA after HU was either treated or not. As performed in Figure 2B, except that the cells were treated or not treated with HU for 20 min. Each panel of results is representative of at least two independently performed transfections followed by MBP pull-down experiments.

We next investigated histone mRNP remodeling using reporter mRNAs either containing or not containing histone stem-loop structure. To this end, we employed bacteriophage MS2 coat protein (MS2) and maltose-binding protein (MBP) system (Figure 2A), by which MS2-binding sites (MS2bs)-containing mRNP can be recovered on amylose beads via the MS2-fused MBP because of the strong and selective interaction between MS2 and MS2bs. We designed two reporter constructs, RLuc-8bs and RLuc-SL-8bs, both of which contained *RLuc* cDNA followed by eight tandem repeats of MS2bs. The RLuc-SL-8bs contained a histone stem-loop structure (SL) lacking U7 snRNP-binding site between *RLuc* cDNA and MS2bs. The resulting RLuc-SL-8bs mRNA would end in poly(A) tail rather than the stem-loop structure, because of the lack of the cleavage signal right after the stem-loop structure. Three effector constructs were also generated: MS2-HA, HA-MBP and MS2-HA-MBP.

The MBP pull-down results showed that (i) HA-MBP and MS2-HA-MBP, but not MS2-HA, were enriched in the MBP pull-down (Figure 2B, top), (ii) RLuc-8bs mRNA and RLuc-SL-8bs mRNA were enriched only in the pull-down of MS2-HA-MBP (Figure 2B, bottom) and (iii) SLBP was detected only when RLuc-SL-8bs mRNA was purified using MS2-HA-MBP (Figure 2B, top), demonstrating specific pull-down of proteins and mRNAs. Next, the levels of copurified endogenous proteins were analyzed using western blotting (Figure 2B, top). Endogenous CBP80, CTIF, eIF3b, eIF4E and eIF4GI copurified with RLuc-8bs mRNA. However, greater amounts of CBP80 and CTIF and less amounts of eIF4E and eIF4GI copurified with RLuc-SL-8bs mRNA, relative to RLuc-8bs mRNA. In addition, comparable levels of copurified eIF3b were observed when RLuc-8bs mRNA and RLuc-SL-8bs mRNA were purified using MS2-HA-MBP. Considering that CBC-bound mRNP is a precursor form of eIF4E-bound mRNP (2), all these data indicate that SLBP binding to the reporter mRNA stabilizes the association of CBP80 and CTIF with mRNA, inhibiting efficient replacement of CBC by eIF4E.

Using the same system, we further tested the effect of HU treatment on histone mRNP remodeling. The results of pull-down experiments using the MS2/MBP system showed that less amounts of CTIF and eIF3b copurified with RLuc-SL-8bs mRNA upon HU treatment (Figure 2C). To the contrary, the associations of CBP80 and SLBP with RLuc-SL-8bs mRNA were not significantly affected, supporting our conclusion that CTIF is released from the SLBP-containing histone mRNPs upon the inhibition of DNA replication.

### ATR and DNA-PK activated by the inhibition of DNA replication promote the release of CTIF from SLBP complex

In this study, we observed that CTIF is released from SLBP-containing complex upon HU treatment (Figures 1 and 2). And it is known that that histone mRNA degradation induced by HU treatment is blocked by treatment with PIKK inhibitors (48,49). Therefore, it is plausible the dissociation of CTIF and SLBP upon HU treatment involves PIKK signaling.

**Figure 3.**
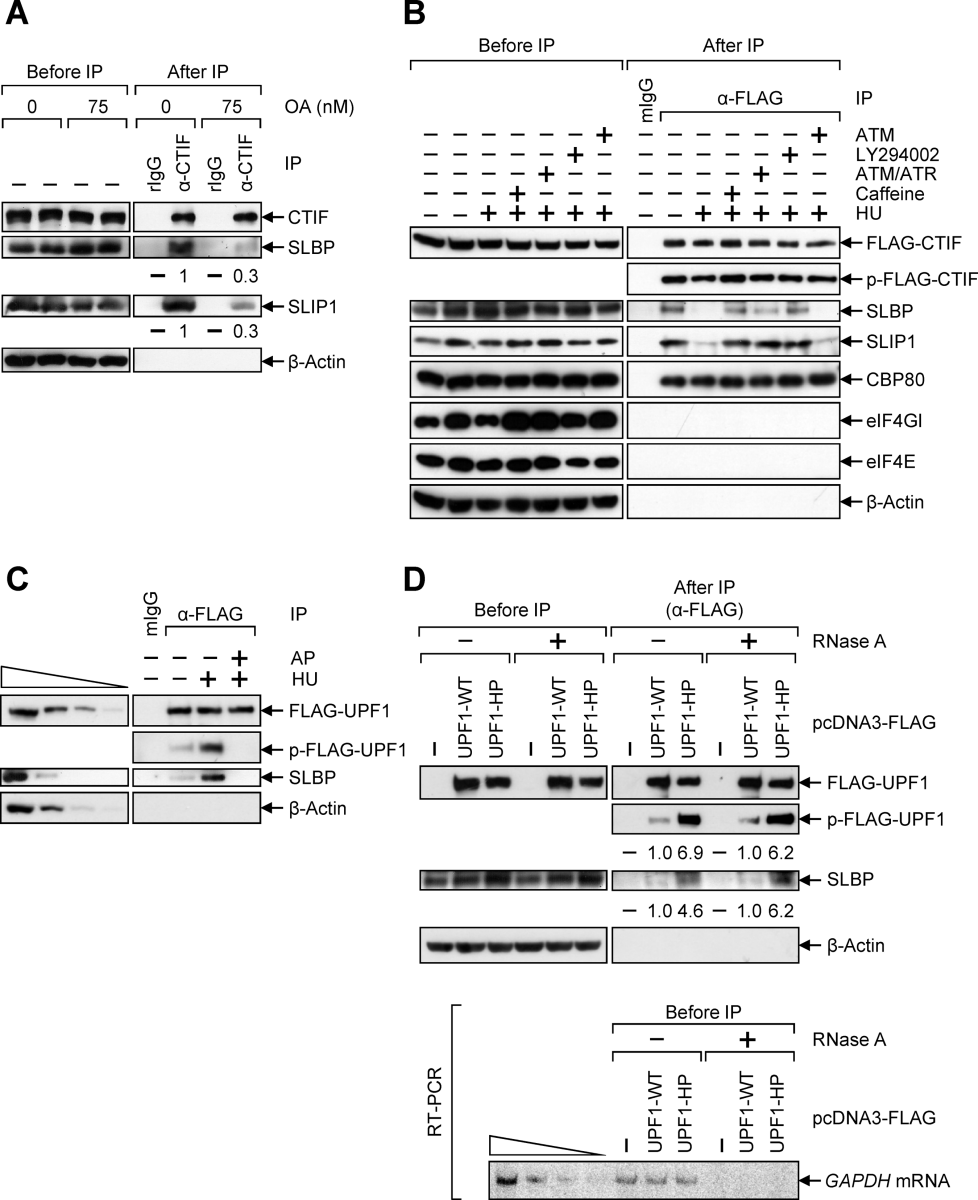
ATR and DNA-PK activated upon the inhibition of DNA replication triggers UPF1 phosphorylation and promotes UPF1–SLBP interaction. (**A**) IP of endogenous CTIF in the presence of OA. HeLa cells were treated with either ethanol (0 nM) or OA (75 nM) for 5 h before IP. Total-cell extracts were analyzed either before or after IP using α-CTIF antibody or rIgG. The levels of co-immunopurified SLBP and SLIP1 were normalized to the levels of immunopurified CTIF. The normalized levels obtained in the IP of CTIF with the treatment of ethanol were arbitrarily set to 1. (**B**) IP of FLAG-CTIF in the presence of PIKK inhibitors. HeLa cells transiently expressing FLAG-CTIF were pre-treated with 15 mM caffeine, 20 μM ATM/ATR inhibitor, 140 μM LY294002 or 1.3 μM ATM inhibitor for 2 h before cell harvest. Cells were either untreated or treated with 5 mM HU for 1 h before cell harvest. IPs were performed using α-FLAG antibody. The level of phosphorylated FLAG-CTIF (p-FLAG-CTIF) was determined by western blotting using α-phospho-S/TQ antibody. The results in panels A and B are representative of at least three independently performed transfections and IPs. (**C**) IP of FLAG-UPF1 using extracts of cells treated with either 5 mM HU or HU/alkaline phosphatase (AP). HeLa cells were transiently transfected with plasmid expressing FLAG-UPF1. Cells were either untreated or treated with 5 mM HU for 1 h before cell harvest. IPs were performed using α-FLAG-conjugated agarose beads. The immunopurified complex was or was not treated with AP. The level of phosphorylated FLAG-UPF1 (p-FLAG-UPF1) was determined by western blotting using α-phospho-S/TQ antibody. To show that western blotting used in this study was sufficiently semi-quantitative, 3-fold serial dilutions of total-cell extracts are represented in the four left-most lanes. (**D**) IP of FLAG-UPF1-WT and FLAG-UPF1-HP. HeLa cells were transiently transfected with plasmid expressing either FLAG, FLAG-UPF1-WT or -HP. Total-cell extracts were either untreated or treated with RNase A and subjected to IP using α-FLAG antibody-conjugated agarose beads. The levels of phosphorylated FLAG-UPF1 (p-FLAG-UPF1) were determined by western blotting using α-phospho-S/TQ antibody (top). The levels of p-FLAG-UPF1 and co-immunopurified SLBP were normalized to the levels of immunopurified FLAG-UPF1. The normalized levels obtained in the IP of FLAG-UPF1-WT were arbitrarily set to 1. Complete removal of cellular RNAs by RNase A treatment was confirmed by RT-PCR using α-[^32^P]-dATP (bottom). To show that the RT-PCR used in this study is sufficiently semi-quantitative, RT-PCR products of 2-fold serially diluted total RNAs were loaded in the four left-most lanes. The results in panels C and D are representative of at least three independently performed transfections and IPs.

The above possibility was demonstrated by several experiments. Treatment of HeLa cells with OA, a potent inhibitor of protein phosphatase 2A (50), inhibited the association of CTIF with SLBP and SLIP1 by 3-fold (Figure 3A), indicating that a protein phosphorylation is involved in the dissociation of CTIF from SLBP and SLIP1. The dissociation between CTIF and SLBP upon HU treatment was selectively blocked in the presence of caffeine, ATM/ATR inhibitor, and LY294002, but not ATM inhibitor (Figure 3B). Caffeine and LY294002 are a general inhibitor for PIKKs (51) and a selective inhibitor for DNA-PK (52), respectively. Consistent with the change of CTIF–SLBP interaction upon treatment of inhibitors, the treatment of cells with caffeine, ATM/ATR inhibitor and LY294002, but not ATM inhibitor, reversed the abundance and the half-life of replication-dependent histone mRNAs, *HIST2H2AA* mRNA and *HIST1H1C* mRNA decreased by HU treatment (Supplementary Figure S3), relative to a replication-independent histone mRNA, *H3F3A* mRNA (53,54). All these observations suggest that activated DNA-PK and/or ATR promotes the dissociation of CTIF and SLBP and consequently triggers rapid degradation of histone mRNAs upon HU treatment.

### UPF1 is phosphorylated and associates with SLBP more strongly upon the inhibition of DNA replication

Previous reports showed that SLBP more strongly interacts with UPF1 in HU-treated cells and that DNA-PK and ATR trigger UPF1 phosphorylation and rapid histone mRNA degradation (48,49). In this study, we found that DNA-PK and/or ATR promote dissociation of CTIF and SLBP (Figure 3) and rapid histone mRNA degradation (Supplementary Figure S3). Therefore, it is plausible that UPF1 phosphorylation by DNA-PK and ATR activated upon HU treatment affects CTIF–SLBP interaction. To test this possibility, we performed a series of IPs followed by western blotting using α-phospho-S/TQ antibody, which recognizes the specific residues (SQ and TQ) of PIKK substrates (55). In addition, the immunoprecipitated complex was either untreated or treated with alkaline phosphatase (AP), which removes all phosphate groups, and then analyzed by western blotting.

In the case of IP with FLAG-SLBP (Supplementary Figure S4A), α-phospho-S/TQ antibody did not react with immunopurified FLAG-SLBP, consistent with a previous report (49), indicating that SLBP is not a target for DNA-PK and ATR. Western blotting results of co-immunoprecipitates revealed that CT components including CBP80, CTIF and eIF3b (which is a common component of both CT and ET), but not ET components including eIF4E and eIF4GI, efficiently associated with SLBP, consistent with a previous report (18). Intriguingly, CTIF and eIF3b were dissociated from SLBP-containing complex upon HU or HU/AP treatment, consistent with results in Figure 1B. Since CTIF directly interacts with eIF3 (4), it is likely that the eIF3 complex is released along with CTIF from the SLBP complex upon HU treatment. On the other hand, CBP80-SLBP interaction was marginally affected upon HU or HU/AP treatment, due to direct interaction between CBP80 and SLBP (45), as observed in Figure 1A and B. Furthermore, UPF1 was enriched in IP of FLAG-SLBP upon HU treatment but not HU/AP treatment, suggesting that UPF1 associates with SLBP more strongly upon HU treatment in a phosphorylation-dependent manner.

Next, the IP results with FLAG-CTIF (Supplementary Figure S4B) revealed that, although CTIF was phosphorylated by PIKK under normal conditions, its phosphorylation was not affected by HU treatment, indicating that CTIF is not a target for DNA-PK and ATR. Western blotting results of co-immunoprecipitates showed that both SLBP and SLIP1 were dissociated from FLAG-CTIF-containing complex upon HU or HU/AP treatment. In addition, consistent with the results in Figure 1A, comparable amount of CBP80 was detected in the IP of FLAG-CTIF in a way that is independent of HU treatment, which is reasonable because CTIF is considered to be involved in CT of most cellular mRNAs (3). As expected, ET components, eIF4E and eIF4GI were not detected in IP of FLAG-CTIF. Of note, the AP-treated FLAG-CTIF exhibited a slightly faster migration and failed to interact with CBP80, implicating the possible role of DNA-PK- and ATR-independent CTIF phosphorylation in its association with CBP80.

The results of the IP with FLAG-UPF1 showed that α-phospho-S/TQ antibody strongly reacted with immunopurified FLAG-UPF1 when the cells were treated with HU (Figure 3C), which supports the previous report (49) showing that UPF1 is a target for DNA-PK and ATR. In addition, more SLBP was enriched in the IP of FLAG-UPF1 upon HU treatment, but not HU/AP treatment (Figure 3C). These observations suggest that UPF1 is phosphorylated by DNA-PK and ATR activated upon HU treatment, and that the phosphorylated form of UPF1 more strongly associates with SLBP.

To further confirm the effect of UPF1 phosphorylation on UPF1–SLBP interaction, IP was carried out using a wild-type (WT) UPF1 and a hyperphosphorylated form of UPF1, UPF1-HP, in which two glycine residues at amino acids 495 and 497 are mutated to arginine and glutamate, respectively (6). Although comparable amounts of FLAG-UPF1-WT and FLAG-UPF1-HP were immunopurified, FLAG-UPF1-HP was ∼6-fold more phosphorylated than FLAG-UPF1-WT (Figure 3D, top). Notably, endogenous SLBP co-immunopurified by 4- to 6-fold more with FLAG-UPF1-HP than with FLAG-UPF1-WT in a RNase A-resistant manner, indicating that SLBP interacts more strongly with hyperphosphorylated UPF1 than hypophosphorylated UPF1. Complete removal of cellular RNAs by RNase A treatment was confirmed by reverse transcription-PCR (RT-PCR) using α-[^32^P]-dATP (Figure 3D, bottom).

### Hyperphosphorylated UPF1 competes with CTIF for binding to SLBP

**Figure 4.**
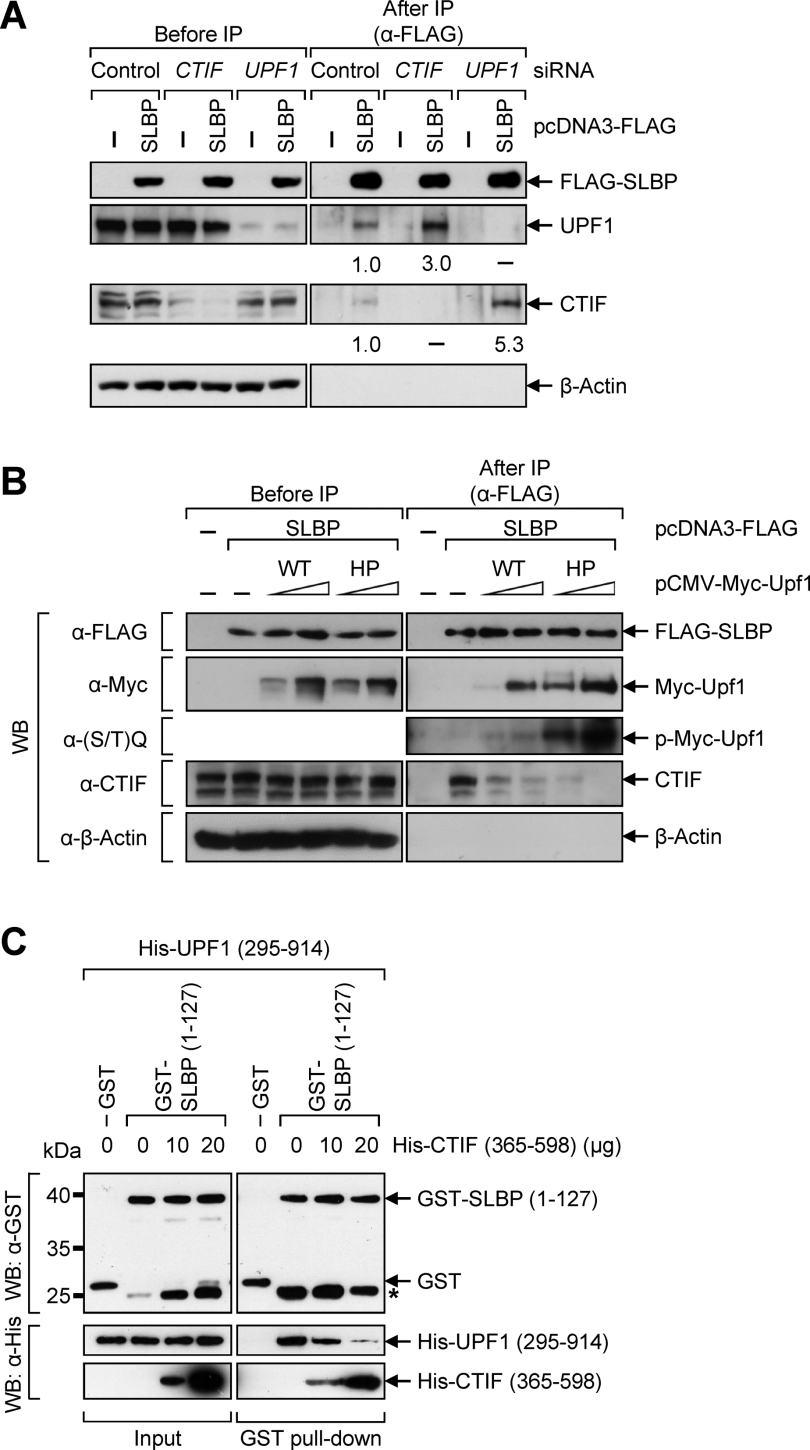
UPF1 competes with CTIF for binding to SLBP. (**A**) HeLa cells were depleted of either CTIF or UPF1 using specific siRNA and then re-transfected with plasmid expressing FLAG-SLBP. Total-cell extracts were analyzed either before or after IP using α-FLAG-conjugated agarose beads. The levels of co-immunopurified cellular UPF1 and CTIF were normalized to the levels of immunopurified FLAG-SLBP. The normalized levels obtained from the IP of FLAG-SLBP in the presence of Control siRNA were set to 1. (**B**) HeLa cells were transiently transfected with the equivalent amount of plasmid expressing FLAG-SLBP and gradually increasing amounts of plasmid expressing either Myc-UPF1-WT or -HP. Total-cell extracts were subjected to IP using α-FLAG-conjugated agarose beads. (**C**) *In vitro* competition assay results. *E. coli* lysates that express GST or GST-SLBP (1–127) were mixed with His-UPF1(295–914) in the presence of gradually increasing amounts of purified His-CTIF (365–598). Purified recombinant bRIP1 (ribosome-inactivating protein 1 from barley seeds), which served as a negative control, was added to the reactions to ensure addition of equal amounts of total protein. After GST pull-down, the purified proteins were analyzed by western blotting (WB) using α-GST antibody (upper) or α-6xHis antibody (lower). The locations of molecular weight (MW) markers are indicated on the left. Asterisk indicates nonspecific bands or degradation products of GST-SLBP (1–127). Each panel of results is representative of at least two independently performed experiments.

Considering that (i) CTIF dissociates from SLBP upon the inhibition of DNA replication or at the end of S phase (Figures 1 and 2) and (ii) histone mRNA degradation requires an interaction between SLBP and UPF1 (48), (iii) hyperphosphorylated UPF1 associates more strongly with SLBP upon HU treatment (Figure 3C and D), it is conceivable that UPF1 competes with CTIF for binding SLBP. This possibility was demonstrated by three approaches: IPs using the extracts of cells depleted of CTIF or UPF1 (Figure 4A), IPs of SLBP in the presence of overexpressed UPF1-WT or -HP (Figure 4B), and *in vitro* GST pull-down experiments using purified recombinant proteins (Figure 4C).

First, downregulation of CTIF using small interfering RNA (siRNA) increased the association of FLAG-SLBP and UPF1 by 3-fold (Figure 4A). In addition, downregulation of UPF1 increased the association of FLAG-SLBP and CTIF by 5.3-fold, indicating competition between CTIF and UPF1 for binding to SLBP.

Second, the association between FLAG-SLBP and endogenous CTIF was gradually inhibited when Myc-UPF1-WT or Myc-UPF1-HP was gradually overexpressed (Figure 4B). Importantly, overexpression of Myc-UPF1-HP more strongly inhibited the CTIF–SLBP interaction, compared to overexpression of Myc-UPF1-WT.

Third, to perform *in vitro* GST pull-down analysis, three recombinant proteins were purified: (i) GST-tagged N-terminal half of SLBP, SLBP(1–127), which contains a translation-activation domain (40,42,46) and are sufficient for binding to CTIF (18) and UPF1 (Supplementary Figure S5), (ii) 6xHis-tagged N-terminally deleted form of CTIF, His-CTIF(365–598), which contains a minimal region for binding SLBP (18) and (iii) 6xHis-tagged UPF1 lacking the N-terminus and C-terminus, His-UPF1(295–914) (25). We failed to obtain recombinant His-tagged full-length UPF1, because recombinant full-length UPF1 was very unstable under our conditions. The results of *in vitro* GST pull-down analysis showed that GST-SLBP(1–127) directly interacted with purified recombinant His-UPF1(295–914) (Figure 4C), suggesting that UPF1(295–914) is sufficient for binding SLBP and the remaining region of UPF1 may be involved in regulation of this interaction. Intriguingly, the interaction between GST-SLBP(1–127) and His-UPF1(295–914) was inhibited by adding increasing amount of recombinant His-CTIF (365–598).

Taken together with the results in Figure 3, our observations suggest that, when DNA replication is inhibited, DNA-PK and ATR are activated and triggers UPF1 hyperphosphorylation. The hyperphosphorylated UPF1 associates with SLBP more strongly and promotes the release of CTIF from SLBP-containing histone mRNPs, leading to translational inhibition and rapid degradation of histone mRNAs.

### PNRC2 and SMG5 are complexed with SLBP in a UPF1 phosphorylation-dependent manner

It is previously known that histone mRNA degradation involves 5′-to-3′ and 3′-to-5′ exoribonucleolytic pathways (37). In particular, oligouridylation of the 3′-end of histone mRNA by terminal uridylyl transferases (TUTases) is critical for the loading of the Lsm1–7 complex (37,56), which recruits a decapping complex composed of DCP1A and DCP2 (57). Given that (i) hyperphosphorylated UPF1 more strongly interacts with SLBP (Figures 3D and 4), (ii) UPF1–SLBP interaction is important for histone mRNA degradation (48,49) and (iii) hyperphosphorylated UPF1 recruits PNRC2, SMG5, SMG6 and SMG7 for efficient NMD as has been reviewed (13–17), therefore we hypothesized that hyperphosphorylated UPF1 may recruit PNRC2, SMG5, SMG6 or SMG7 for histone mRNA degradation in a way that is either coupled or not to TUTases recruitment.

**Figure 5.**
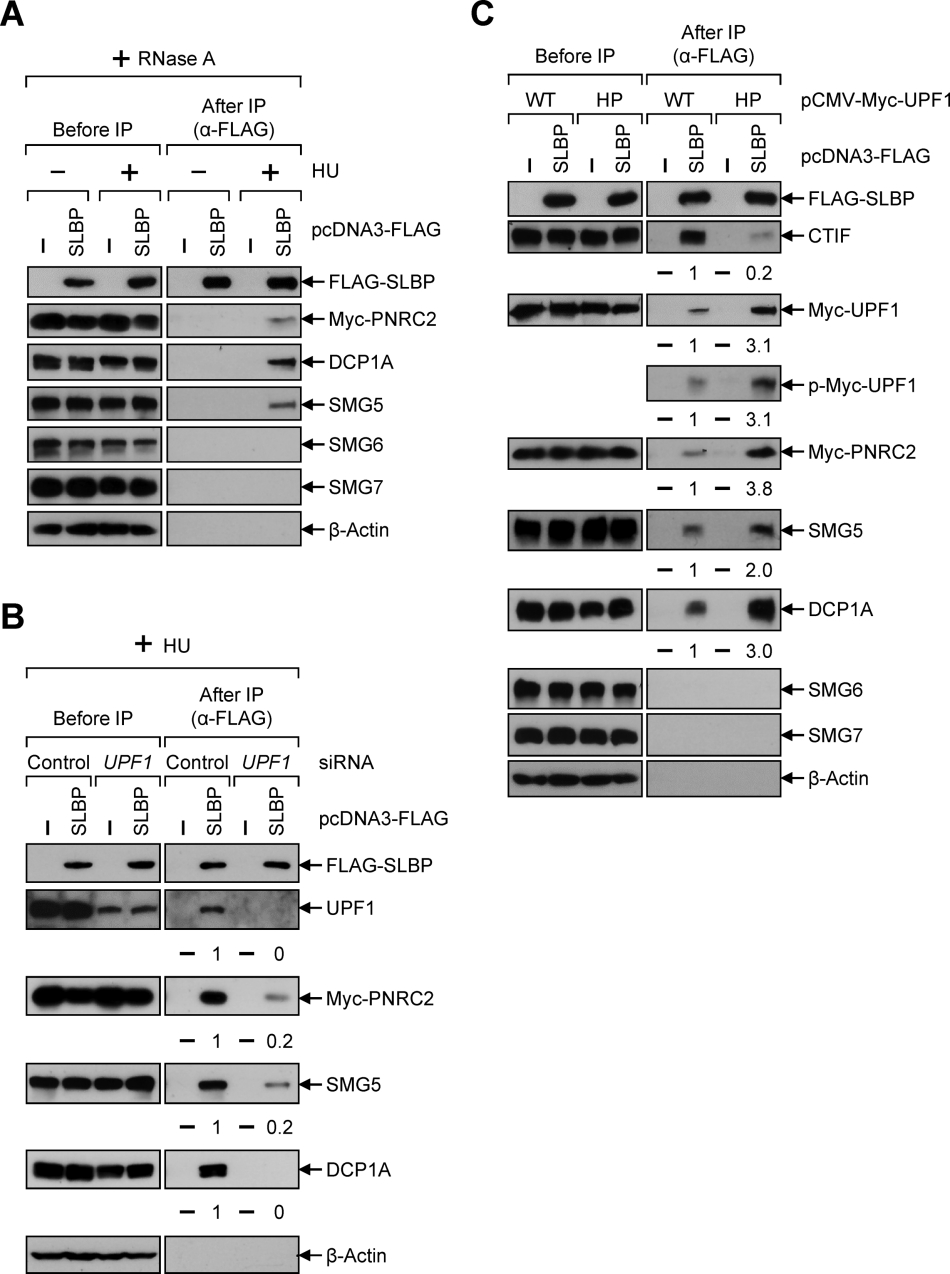
PNRC2 and SMG5 associate with SLBP in a way that depends on UPF1 phosphorylation. (**A**) HEK293T cells were transiently transfected with plasmids expressing FLAG-SLBP and Myc-PNRC2. Cells were either treated or not treated with 5 mM HU for 20 min before cell harvest. Total-cell extracts were treated with RNase A and subjected to IP using α-FLAG-conjugated agarose beads. (**B**) HEK293T cells were transiently transfected with either *UPF1* siRNA or nonspecific control siRNA. Two days later, cells were retransfected with plasmids expressing FLAG-SLBP and Myc-PNRC2. Cells were treated with HU for 20 min before cell harvest. Total-cell extracts were treated with RNase A and subjected to IP using α-FLAG-conjugated agarose beads. The levels of co-immunopurified proteins were normalized to the levels of immunopurified FLAG-SLBP. The normalized levels obtained in the IP of FLAG-SLBP in the presence of control siRNA were arbitrarily set to 1. Each panel of results is representative of at least two independently performed transfections and IPs. (**C**) As performed in Figure 5B, except that HEK293T cells were transfected with plasmids expressing FLAG-SLBP and either Myc-UPF1-WT or Myc-UPF1-HP. Each panel of results is representative of at least two independently performed transfections and IPs.

To test the above possibility, we first investigated the possible association between SLBP and UPF1-interacting factors (PNRC2 and SMG5–7) using IPs. The results showed that PNRC2 and SMG5, but not SMG6 and SMG7, were preferentially enriched in the IP of SLBP in a RNase A-resistant and HU-dependent manner (Figure 5A), indicating that SLBP is preferentially complexed with PNRC2 and SMG5. Consistent with the direct interaction between PNRC2 and DCP1A (25), Myc-PNRC2 and endogenous DCP1A were observed in IP of SLBP, supporting a 5′-to-3′ degradation of histone mRNAs (48,58).

Our observation that SLBP associates with PNRC2 and SMG5 (Figure 5A) led us to test whether UPF1 functions to bridge SLBP and PNRC2/SMG5 complex in histone mRNA degradation. This was evident by downregulation-coupled IPs. The results showed that specific downregulation of UPF1 using siRNA drastically inhibited association of SLBP with Myc-PNRC2, SMG5 and DCP1A (Figure 5B). These data indicate that UPF1 functions as an adaptor to link SLBP and PNRC2/SMG5.

We next asked if the recruitment of PNRC2, SMG5 and DCP1A to SLBP-containing complex is dependent on UPF1 phosphorylation. To this end, cells were overexpressed with either UPF1-WT or UPF1-HP. And then the change in the composition of SLBP-containing complex was assessed by IPs. The results showed that greater amounts of PNRC2, SMG5 and DCP1A co-immunopurified with FLAG-SLBP when cells were overexpressed with UPF1-HP, compared with UPF1-WT (Figure 5C). These data suggest that UPF1 phosphorylation promotes the recruitment of PNRC2, SMG5 and DCP1A to SLBP-containing histone mRNP.

### PNRC2 and SMG5 are functionally involved in replication-dependent histone mRNA degradation

**Figure 6.**
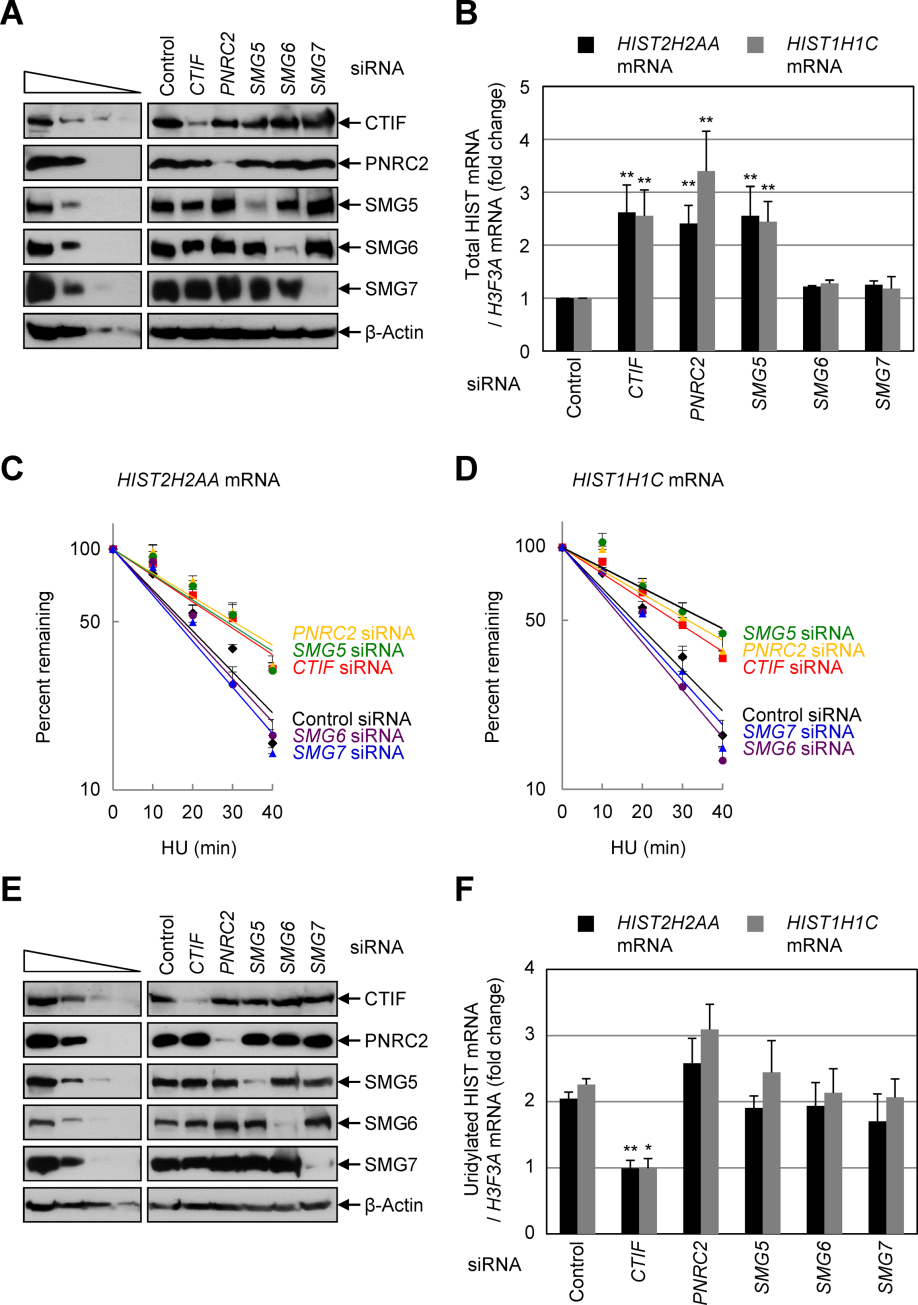
Downregulation of PNRC2 or SMG5 increases the levels and half-lives of replication-dependent histone mRNAs without affecting oligouridylation. (**A** and **B**) HeLa cells were transiently transfected with the indicated siRNA. Three days later, cells were treated with 5 mM HU for 1 h. Total-cell proteins and RNAs were purified. (**A**) Western blotting demonstrating specific downregulation by siRNA transfection. (**B**) qRT-PCR of *HIST2H2AA* mRNAs, *HIST1H1C* mRNAs and *H3F3A* mRNAs. The levels of replication-dependent histone mRNAs, *HIST2H2AA* mRNAs and *HIST1H1C* mRNAs were normalized to the levels of replication-independent histone mRNA, *H3F3A* mRNAs. The relative ratios of normalized levels obtained from cells treated with HU to those obtained from cells untreated with HU in the presence of each siRNA were calculated. The relative ratio (fold-change) obtained in the presence of control siRNA was arbitrarily set to 1. (**C** and **D**) Half-lives of *HIST2H2AA* mRNAs and *HIST1H1C* mRNAs. HeLa cells were transiently transfected with the indicated siRNA. Three days later, cells were treated with 5 mM HU. Total-cell RNAs were purified at the indicated time points after HU treatment. The levels of *HIST2H2AA* mRNAs (**C**) or *HIST1H1C* mRNAs (**D**) were normalized to the levels of *H3F3A* mRNA. The normalized levels of *HIST2H2AA* mRNAs (**C**) or *HIST1H1C* mRNAs (**D**) were plotted as a function of time after HU treatment. The symbols and bars in each panel represent the mean and standard deviation of at least three independently performed transfections and qRT-PCRs. (**E** and **F**) As in Figure 6A and B, except that cells were treated with HU for 20 min. (**E**) Western blotting demonstrating specific downregulation by siRNA transfection. (**F**) qRT-PCR of oligouridylated *HIST2H2AA* mRNAs and *HIST1H1C* mRNAs. The levels of oligouridylated *HIST2H2AA* mRNAs and *HIST1H1C* mRNAs were normalized to the levels of *H3F3A* mRNAs. The ratios of normalized levels obtained from cells treated with HU to those obtained from cells untreated with HU in the presence of each siRNA were calculated. The columns and bars in each panel represent the mean and standard deviation of at least three independently performed transfections and qRT-PCRs. **P < 0.01, *P < 0.05 as determined by Student's t-test.

The results in Figure 5 suggested that the recruitment of PNRC2/SMG5 to histone mRNP via UPF1 triggers histone mRNA degradation. To clearly demonstrate this possibility, HeLa cells were depleted of PNRC2, SMG5, SMG6 or SMG7 and the level of histone mRNAs was measured by qRT-PCR. As a positive control, CTIF was downregulated, since CTIF downregulation inhibits efficient CT and consequently blocks rapid degradation of histone mRNAs (18). The specific downregulations by siRNAs were confirmed by western blotting (Figure 6A). The qRT-PCR results showed that downregulation of CTIF, PNRC2 and SMG5, but not SMG6 and SMG7, significantly increased the abundance (Figure 6B) and half-lives (Figure 6C and D) of *HIST2H2AA* mRNA and *HIST1H1C* mRNA, indicating that PNRC2 and SMG5 are involved in histone mRNA degradation when DNA replication is inhibited.

It is previously shown that the decapping of histone mRNA is mediated by oligouridylation of the 3′-end of histone mRNA, loading of the Lsm1–7 complex onto oligouridine, and recruitment of the decapping complex (48). In addition, our results suggested the existence of an additional pathway for 5′-to-3′ degradation of histone mRNAs. Therefore, we next investigated the possible crosstalk between the oligouridylation-mediated 5′-to-3′ degradation and the PNRC2/SMG5-mediated 5′-to-3′ degradation of histone mRNAs. To this end, we compared the level of oligouridylated histone mRNAs upon PNRC2 or SMG5 downregulation. Western blotting demonstrated the specific downregulation by siRNAs (Figure 6E). qRT-PCR results showed that, consistent with our previous report (18), downregulation of CTIF completely blocked the HU treatment-induced oligouridylation of *HIST2H2AA* mRNAs and *HIST1H1C* mRNAs (Figure 6F). This is reasonable because CTIF is necessary for histone mRNA translation, which precedes histone mRNA degradation. To the contrary, downregulation of PNRC2 or SMG5 did not significantly affect the level of oligouridylated histone mRNAs (Figure 6F), suggesting that PNRC2/SMG5-mediated 5′-to-3′ degradation of histone mRNAs occurs independently of the oligouridylation of the 3′-end of histone mRNA. In support of this conclusion, double downregulation of TUTase 1 and 3, which are involved in oligouridylation of histone mRNA (37), had no significant effect on the composition of SLBP-containing complex (Supplementary Figure S6).

## DISCUSSION

**Figure 7.**
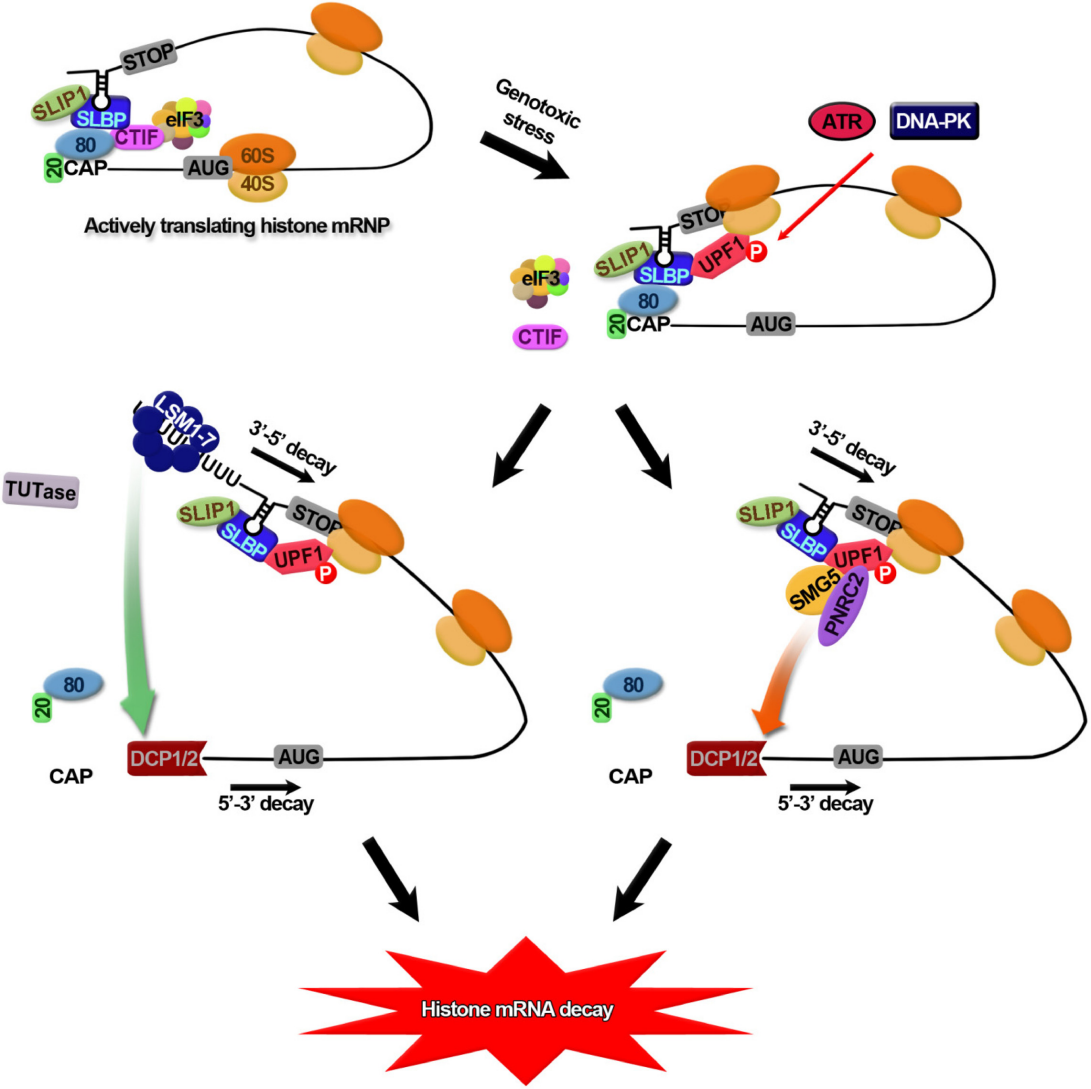
The proposed model for the histone mRNP remodeling occurring during switching from active CT and rapid mRNA degradation. The details are described in the discussion section. AUG, translation initiation codon; STOP, translation termination codon; 20, CBP20; 80, CBP80; 40, 40S ribosome subunit; 60, 60S ribosome subunit.

In this study, we investigate the remodeling of replication-dependent histone mRNP occurring during the switch from an actively translating mode to an mRNA degrading mode (Figure 7). A majority of histone mRNAs are actively translated in the cytoplasm with CBC bound to the 5′-end (18) and with SLBP bound to the 3′-end of histone mRNAs (19,20). Both CBC (3) and SLBP (18) have a common ability to recruit CTIF, possibly inducing a circularization of histone mRNPs. In support of this possibility, direct interaction between CBP80 and SLBP has been also reported (45). With the help of these multiple cross-interactions, the circularized histone mRNP would be stabilized. It is unknown which protein, CBP80 and/or SLBP, is primarily involved in the recruitment of CTIF. Nonetheless, the recruited CTIF can direct the efficient CT of histone mRNAs (18), since (i) CTIF directly interacts with eIF3 complex via eIF3g (3,4) and (ii) tethered CTIF promotes translation of a downstream open reading frame (4).

When the cells encounter DNA replication stress, histone mRNPs undergo drastic remolding, which is favorable to mRNA degradation. Either ATR or DNA-PK or both are activated, leading to hyperphosphorylation of UPF1 (Figure 3). UPF1 hyperphosphorylated by ATR or DNA-PK associates with SLBP more strongly than does CTIF (Figures 3D and 4), resulting in the release of CTIF and CTIF-associating eIF3 from SLBP-containing complex (Figures 1B and 5; Supplementary Figure S4). As a result, the CT of histone mRNA would be inhibited and the hyperphosphorylated UPF1 may recruit either TUTases by an unknown mechanism (37) and/or PNRC2/SMG5 (in this study) via a direct interaction between UPF1 and PNRC2 (25). The recruited TUTases triggers the oligouridylation of the 3′-end of histone mRNAs and then the Lsm1–7 complex is loaded onto the oligouridylated sequences (48). Eventually, the Lsm1–7 recruits decapping complex (57). As an alternative pathway, the recruited PNRC2/SMG5 may directly interact with decapping complex (25) without affecting oligouridylation (Figure 6). The decapping complex joined through two independent routes would cleave cap structure triggering 5′-to-3′ degradation of histone mRNAs (Figure 7). Histone mRNAs could be also degraded in a 3′-to-5′ direction by exosome (48,56).

In summary, we demonstrate that the remodeling of histone mRNPs from CTIF-containing SLBP complex to UPF1-containing SLBP complex is critical for the switch from efficient CT to rapid histone mRNA degradation.

## SUPPLEMENTARY DATA

Supplementary Data are available at NAR Online.

SUPPLEMENTARY DATA

## References

[1] Maquat L.E., Tarn W.Y., Isken O. (2010). The pioneer round of translation: features and functions. Cell.

[2] Lejeune F., Ishigaki Y., Li X., Maquat L.E. (2002). The exon junction complex is detected on CBP80-bound but not eIF4E-bound mRNA in mammalian cells: dynamics of mRNP remodeling. EMBO J..

[3] Kim K.M., Cho H., Choi K., Kim J., Kim B.W., Ko Y.G., Jang S.K., Kim Y.K. (2009). A new MIF4G domain-containing protein, CTIF, directs nuclear cap-binding protein CBP80/20-dependent translation. Genes Dev..

[4] Choe J., Oh N., Park S., Lee Y.K., Song O.K., Locker N., Chi S.G., Kim Y.K. (2012). Translation initiation on mRNAs bound by nuclear cap-binding protein complex CBP80/20 requires interaction between CBP80/20-dependent translation initiation factor and eukaryotic translation initiation factor 3g. J. Biol. Chem..

[5] Ishigaki Y., Li X., Serin G., Maquat L.E. (2001). Evidence for a pioneer round of mRNA translation: mRNAs subject to nonsense-mediated decay in mammalian cells are bound by CBP80 and CBP20. Cell.

[6] Isken O., Kim Y.K., Hosoda N., Mayeur G.L., Hershey J.W., Maquat L.E. (2008). Upf1 phosphorylation triggers translational repression during nonsense-mediated mRNA decay. Cell.

[7] Sharma A., Yilmaz A., Marsh K., Cochrane A., Boris-Lawrie K. (2012). Thriving under Stress: Selective Translation of HIV-1 Structural Protein mRNA during Vpr-Mediated Impairment of eIF4E Translation Activity. PLoS Pathog..

[8] Apcher S., Daskalogianni C., Lejeune F., Manoury B., Imhoos G., Heslop L., Fahraeus R. (2011). Major source of antigenic peptides for the MHC class I pathway is produced during the pioneer round of mRNA translation. Proc. Natl Acad. Sci. U.S.A..

[9] Sato H., Maquat L.E. (2009). Remodeling of the pioneer translation initiation complex involves translation and the karyopherin importin beta. Genes Dev..

[10] Pestova T.V., Lorsch J.R., Hellen C.U., Mathews M.B., Sonenberg N., Hershey J.W.B. (2007). The mechanism of translation initiation in eukaryotes. Translational Control in Biology and Medicine.

[11] Durand S., Lykke-Andersen J. (2013). Nonsense-mediated mRNA decay occurs during eIF4F-dependent translation in human cells. Nat. Struct. Mol. Biol..

[12] Rufener S.C., Muhlemann O. (2013). eIF4E-bound mRNPs are substrates for nonsense-mediated mRNA decay in mammalian cells. Nat. Struct. Mol. Biol..

[13] Schoenberg D.R., Maquat L.E. (2012). Regulation of cytoplasmic mRNA decay. Nat .Rev. Genet..

[14] Hwang J., Kim Y.K. (2013). When a ribosome encounters a premature termination codon. BMB Rep..

[15] Schweingruber C., Rufener S.C., Zund D., Yamashita A., Muhlemann O. (2013). Nonsense-mediated mRNA decay - Mechanisms of substrate mRNA recognition and degradation in mammalian cells.

[16] Popp M.W., Maquat L.E. (2013). Organizing principles of Mammalian nonsense-mediated mRNA decay. Ann. Rev. Genet..

[17] Karam R., Wengrod J., Gardner L.B., Wilkinson M.F. (2013). Regulation of nonsense-mediated mRNA decay: implications for physiology and disease. Biochim. Biophys. Acta.

[18] Choe J., Kim K.M., Park S., Lee Y.K., Song O.K., Kim M.K., Lee B.G., Song H.K., Kim Y.K. (2013). Rapid degradation of replication-dependent histone mRNAs largely occurs on mRNAs bound by nuclear cap-binding proteins 80 and 20. Nucleic Acids Res..

[19] Jaeger S., Barends S., Giege R., Eriani G., Martin F. (2005). Expression of metazoan replication-dependent histone genes. Biochimie.

[20] Marzluff W.F., Wagner E.J., Duronio R.J. (2008). Metabolism and regulation of canonical histone mRNAs: life without a poly(A) tail. Nat. Rev. Genet..

[21] Hoefig K.P., Heissmeyer V. (2014). Degradation of oligouridylated histone mRNAs: see UUUUU and goodbye.

[22] Tan D., Marzluff W.F., Dominski Z., Tong L. (2013). Structure of histone mRNA stem-loop, human stem-loop binding protein, and 3’hExo ternary complex. Science.

[23] Lovejoy C.A., Cortez D. (2009). Common mechanisms of PIKK regulation. DNA Repair (Amst.).

[24] Cho H., Han S., Choe J., Park S.G., Choi S.S., Kim Y.K. (2013). SMG5-PNRC2 is functionally dominant compared with SMG5-SMG7 in mammalian nonsense-mediated mRNA decay. Nucleic Acids Res..

[25] Cho H., Kim K.M., Kim Y.K. (2009). Human proline-rich nuclear receptor coregulatory protein 2 mediates an interaction between mRNA surveillance machinery and decapping complex. Mol. Cell.

[26] Chiu S.Y., Serin G., Ohara O., Maquat L.E. (2003). Characterization of human Smg5/7a: a protein with similarities to Caenorhabditis elegans SMG5 and SMG7 that functions in the dephosphorylation of Upf1. RNA.

[27] Gatfield D., Unterholzner L., Ciccarelli F.D., Bork P., Izaurralde E. (2003). Nonsense-mediated mRNA decay in Drosophila: at the intersection of the yeast and mammalian pathways. EMBO J..

[28] Unterholzner L., Izaurralde E. (2004). SMG7 acts as a molecular link between mRNA surveillance and mRNA decay. Mol. Cell.

[29] Fukuhara N., Ebert J., Unterholzner L., Lindner D., Izaurralde E., Conti E. (2005). SMG7 is a 14–3–3-like adaptor in the nonsense-mediated mRNA decay pathway. Mol. Cell.

[30] Cho H., Kim K.M., Han S., Choe J., Park S.G., Choi S.S., Kim Y.K. (2012). Staufen1-mediated mRNA decay functions in adipogenesis. Mol. Cell.

[31] Kim Y.K., Furic L., Desgroseillers L., Maquat L.E. (2005). Mammalian Staufen1 recruits Upf1 to specific mRNA 3’UTRs so as to elicit mRNA decay. Cell.

[32] Zhang J., Sun X., Qian Y., Maquat L.E. (1998). Intron function in the nonsense-mediated decay of beta-globin mRNA: indications that pre-mRNA splicing in the nucleus can influence mRNA translation in the cytoplasm. RNA.

[33] Moriarty P.M., Reddy C.C., Maquat L.E. (1998). Selenium deficiency reduces the abundance of mRNA for Se-dependent glutathione peroxidase 1 by a UGA-dependent mechanism likely to be nonsense codon-mediated decay of cytoplasmic mRNA. Mol. Cell. Biol..

[34] Belgrader P., Cheng J., Zhou X., Stephenson L.S., Maquat L.E. (1994). Mammalian nonsense codons can be cis effectors of nuclear mRNA half-life. Mol. Cell. Biol..

[35] Nott A., Le Hir H., Moore M.J. (2004). Splicing enhances translation in mammalian cells: an additional function of the exon junction complex. Genes Dev..

[36] Cho H., Han S., Park O.H., Kim Y.K. (2013). SMG1 regulates adipogenesis via targeting of staufen1-mediated mRNA decay. Biochim. Biophys. Acta.

[37] Mullen T.E., Marzluff W.F. (2008). Degradation of histone mRNA requires oligouridylation followed by decapping and simultaneous degradation of the mRNA both 5’ to 3’ and 3’ to 5’. Genes Dev..

[38] Niranjanakumari S., Lasda E., Brazas R., Garcia-Blanco M.A. (2002). Reversible cross-linking combined with immunoprecipitation to study RNA-protein interactions in vivo. Methods.

[39] Zhao X., McKillop-Smith S., Muller B. (2004). The human histone gene expression regulator HBP/SLBP is required for histone and DNA synthesis, cell cycle progression and cell proliferation in mitotic cells. J. Cell Sci..

[40] Cakmakci N.G., Lerner R.S., Wagner E.J., Zheng L., Marzluff W.F. (2008). SLIP1, a factor required for activation of histone mRNA translation by the stem-loop binding protein. Mol. Cell. Biol..

[41] Shyu Y.J., Liu H., Deng X., Hu C.D. (2006). Identification of new fluorescent protein fragments for bimolecular fluorescence complementation analysis under physiological conditions. Biotechniques.

[42] Kaygun H., Marzluff W.F. (2005). Translation termination is involved in histone mRNA degradation when DNA replication is inhibited. Mol. Cell. Biol..

[43] Graves R.A., Pandey N.B., Chodchoy N., Marzluff W.F. (1987). Translation is required for regulation of histone mRNA degradation. Cell.

[44] Baumbach L.L., Marashi F., Plumb M., Stein G., Stein J. (1984). Inhibition of DNA replication coordinately reduces cellular levels of core and H1 histone mRNAs: requirement for protein synthesis. Biochemistry.

[45] Narita T., Yung T.M., Yamamoto J., Tsuboi Y., Tanabe H., Tanaka K., Yamaguchi Y., Handa H. (2007). NELF interacts with CBC and participates in 3’ end processing of replication-dependent histone mRNAs. Mol. Cell.

[46] Sanchez R., Marzluff W.F. (2002). The stem-loop binding protein is required for efficient translation of histone mRNA in vivo and in vitro. Mol. Cell. Biol..

[47] Bansal N., Zhang M., Bhaskar A., Itotia P., Lee E., Shlyakhtenko L.S., Lam T.T., Fritz A., Berezney R., Lyubchenko Y.L. (2013). Assembly of the SLIP1-SLBP Complex on Histone mRNA Requires Heterodimerization and Sequential Binding of SLBP Followed by SLIP1. Biochemistry.

[48] Kaygun H., Marzluff W.F. (2005). Regulated degradation of replication-dependent histone mRNAs requires both ATR and Upf1. Nat. Struct. Mol. Biol..

[49] Muller B., Blackburn J., Feijoo C., Zhao X., Smythe C. (2007). DNA-activated protein kinase functions in a newly observed S phase checkpoint that links histone mRNA abundance with DNA replication. J. Cell Biol..

[50] Haystead T.A., Sim A.T., Carling D., Honnor R.C., Tsukitani Y., Cohen P., Hardie D.G. (1989). Effects of the tumour promoter okadaic acid on intracellular protein phosphorylation and metabolism. Nature.

[51] Sarkaria J.N., Busby E.C., Tibbetts R.S., Roos P., Taya Y., Karnitz L.M., Abraham R.T. (1999). Inhibition of ATM and ATR kinase activities by the radiosensitizing agent, caffeine. Cancer Res..

[52] Stiff T., O'Driscoll M., Rief N., Iwabuchi K., Lobrich M., Jeggo P.A. (2004). ATM and DNA-PK function redundantly to phosphorylate H2AX after exposure to ionizing radiation. Cancer Res..

[53] Wells D., Kedes L. (1985). Structure of a human histone cDNA: evidence that basally expressed histone genes have intervening sequences and encode polyadenylylated mRNAs. Proc. Natl Acad. Sci. U.S.A..

[54] Brush D., Dodgson J.B., Choi O.R., Stevens P.W., Engel J.D. (1985). Replacement variant histone genes contain intervening sequences. Mol. Cell. Biol..

[55] O'Neill T., Dwyer A.J., Ziv Y., Chan D.W., Lees-Miller S.P., Abraham R.H., Lai J.H., Hill D., Shiloh Y., Cantley L.C. (2000). Utilization of oriented peptide libraries to identify substrate motifs selected by ATM. J. Biol. Chem..

[56] Slevin M.K., Meaux S., Welch J.D., Bigler R., Miliani de Marval P.L., Su W., Rhoads R.E., Prins J.F., Marzluff W.F. (2014). Deep sequencing shows multiple oligouridylations are required for 3’ to 5’ degradation of histone mRNAs on polyribosomes. Mol. Cell.

[57] Coller J.M., Tucker M., Sheth U., Valencia-Sanchez M.A., Parker R. (2001). The DEAD box helicase, Dhh1p, functions in mRNA decapping and interacts with both the decapping and deadenylase complexes. RNA.

[58] Su W., Slepenkov S.V., Slevin M.K., Lyons S.M., Ziemniak M., Kowalska J., Darzynkiewicz E., Jemielity J., Marzluff W.F., Rhoads R.E. (2013). mRNAs containing the histone 3’ stem-loop are degraded primarily by decapping mediated by oligouridylation of the 3’ end. RNA.

