# Supplementary material for: The mRNP remodeling mediated by UPF1 promotes rapid degradation of replication-dependent histone mRNA
Source: Nucleic Acids Res. 2014 Jul 12;42(14):9334–49. doi: 10.1093/nar/gku610 (PMC4132728; doi:10.1093/nar/gku610)
Supplement: SUPPLEMENTARY DATA [file supp_42_14_9334__index.html]

The mRNP remodeling mediated by UPF1 promotes rapid degradation of replication-dependent histone mRNA — The mRNP remodeling mediated by UPF1 promotes rapid degradation of replication-dependent histone mRNA — SUPPLEMENTARY DATA 

# The mRNP remodeling mediated by UPF1 promotes rapid degradation of replication-dependent histone mRNA

## SUPPLEMENTARY DATA

**Files in this Data Supplement:**

- SUPPLEMENTARY DATA
