# Supplementary material for: The mRNP remodeling mediated by UPF1 promotes rapid degradation of replication-dependent histone mRNA
Source: Nucleic Acids Res. 2014 Jul 12;42(14):9334–49. doi: 10.1093/nar/gku610 (PMC4132728; doi:10.1093/nar/gku610)
Supplement: SUPPLEMENTARY DATA [file supp_gku610_nar-00722-m-2014-File009.pdf]

SUPPLEMENTARY DATA

The mRNP remodeling mediated by UPF1 promotes rapid degradation of replication-dependent histone mRNA

Junho Choe[1], Sang Ho Ahn[1], and Yoon Ki Kim[1,*]

[1]Division of Life Sciences, Korea University, Seoul 136-701, Republic of Korea

[*]To whom correspondence should be addressed. E-mail: yk-kim@korea.ac.kr; Tel: 82-2-3290-3410; FAX: 82-2-923-9923 (Y.K.K).

Running title: Histone mRNP remodeling during mRNA degradation

Key words: Histone mRNA; CTIF; SLBP; CBP80; UPF1; PIKK; NMD

Supplementary Figure Legends

Supplementary Figure S1. An introduction of the W75A substitution in SLBP causes the release of CTIF and eIF3 components from SLBP-containing complex. HEK293T cells were transiently transfected with plasmid expressing either FLAG-SLBP-WT or -W75A. Total-cell extracts were treated with RNase A and then subjected to IP using α-FLAG-conjugated agarose beads. Protein samples before and after IP were analyzed by Western blotting.

Supplementary Figure S2. CTIF-SLIP1 interaction is disrupted upon HU treatment. (**A**) IPs of FLAG-SLIP1**.** HEK293T cells were transiently transfected with a plasmid expressing either FLAG or FLAG-SLIP1. Total-cell extracts were treated with RNase A and subjected to IP using α-FLAG-conjugated agarose beads. (**B**) IPs of FLAG-SLIP1 using extracts of cells either untreated or treated with HU. As in Supplementary Figure S2A, except that cells were either untreated or treated with HU for 1 h before cell harvest.

Supplementary Figure S3. Rapid degradation of histone mRNA upon HU treatment is abolished in the presence of ATR or DNA-PK inhibitor. (**A**) HeLa cells were untreated or pre-treated with caffeine for 2 h before cell harvest. Additionally, cells were either untreated or treated with HU for 40 min before cell harvest. The levels of *HIST2H2AA* mRNAs and *H3F3A* mRNAs were analyzed by qRT-PCR. The levels of *HIST2H2AA* mRNAs were normalized to the levels of *H3F3A* mRNA. The normalized levels of *HIST2H2AA* mRNAs in the absence of HU were then set to 100%. (**B**) As performed in Supplementary Figure S3A, except that the levels of *HIST1H1C* mRNAs were analyzed

by qRT-PCR. (**C**) HeLa cells were untreated or pre-treated with 1-, 10-, or 100-fold of the IC_{50} concentration of various PIKK inhibitors (IC_{50} = 13 nM for ATM inhibitor, IC_{50} = 200 nM for ATM/ATR inhibitor, and IC_{50} = 1.4 μM for LY294002) for 2 h before cell harvest. The cells were also either untreated or treated with HU for 40 min before cell harvest. The levels of *HIST2H2AA* mRNAs and *H3F3A* mRNAs were analyzed by qRT-PCR. The levels of *HIST2H2AA* mRNAs were normalized to the levels of *H3F3A* mRNAs. The normalized level of *HIST2H2AA* mRNAs in the absence of HU was then set to 100%. (**D**) As performed in Supplementary Figure S3C, except that the levels of *HIST1H1C* mRNAs were analyzed by qRT-PCR. (**E**) Half-life of *HIST2H2AA* mRNAs. The levels of *HIST2H2AA* mRNAs were normalized to the level of *H3F3A* mRNAs. The normalized levels of *HIST2H2AA* mRNAs obtained from were plotted as a function of time after HU treatment. Normalized levels of *HIST2H2AA* mRNAs obtained at 0 min after HU treatment in the presence of each inhibitor were arbitrarily set to 100%. (**F**) Half-life of *HIST1H1C* mRNAs. As performed in Supplementary Figure S3E, except that *HIST1H1C* mRNAs were analyzed. The symbols and bars in each panel represent the mean and standard deviation of at least four independently performed transfections and qRT-PCRs. **, $P < 0.01$; *, $P < 0.05$ as determined by Student's t-test.

Supplementary Figure S4. Neither SLBP nor CTIF was phosphorylated in a HU treatment-dependent manner. (**A**) IP of FLAG-SLBP using extracts of cells treated with either HU or HU/AP. As in Figure 3C, except that HeLa cells were transiently transfected with plasmid expressing FLAG-SLBP. p-FLAG-SLBP1, phosphorylated form of FLAG-SLBP. (**B**) IP of FLAG-CTIF using extracts of cells treated with either

HU or HU/AP. As in Figure 3C, except that HeLa cells were transiently transfected with plasmid expressing FLAG-CTIF. p-FLAG-CTIF indicates a phosphorylated form of FLAG-CTIF. Each panel of results is representative of at least three independently performed transfections and IPs.

Supplementary Figure S5. An N-terminal half of SLBP is sufficient for Upf1 binding. HEK293T cells were transiently transfected with plasmid expressing FLAG-Upf1 and either Myc-SLBP or its deletion variant. Total-cell extracts were treated with RNase A and subjected to IP using α-Myc antibody.

Supplementary Figure S6. Double downregulation of TUTase 1 and 3 had no significant effect on the protein composition in SLBP-containing histone mRNP. HEK293T cells were either nondepleted or codepleted of TUTase 1 and 3. Two days later, cells were retransfected with plasmids expressing FLAG-SLBP and Myc-PNRC2. Cells were treated with HU for 20 min before cell harvest. Total-cell extracts were treated with RNase A and subjected to IP using α-FLAG-conjugated agarose beads. (**A**) Western blots of protein samples before and after IPs. (**B**) qRT-PCR of TUTase 1 and 3 mRNAs. Total-cell RNAs were purified before RNase treatment and after HU treatment. The levels of TUTase 1 and 3 mRNAs were normalized to the levels of β-Actin mRNAs. The normalized levels in the presence of control siRNA were arbitrarily set to 100. (**C**) qRT-PCR of *HIST2H2AA* mRNAs or *HIST1H1C* mRNAs. Total-cell RNAs were purified before RNase treatment and after HU treatment. The levels of *HIST2H2AA* mRNAs or *HIST1H1C* mRNAs were normalized to the levels of β-Actin mRNAs. The columns and bars in each panel represent the mean and standard deviation of at least

two independently performed transfections and qRT-PCRs. **, $P < 0.01$ as determined by Student's t-test.

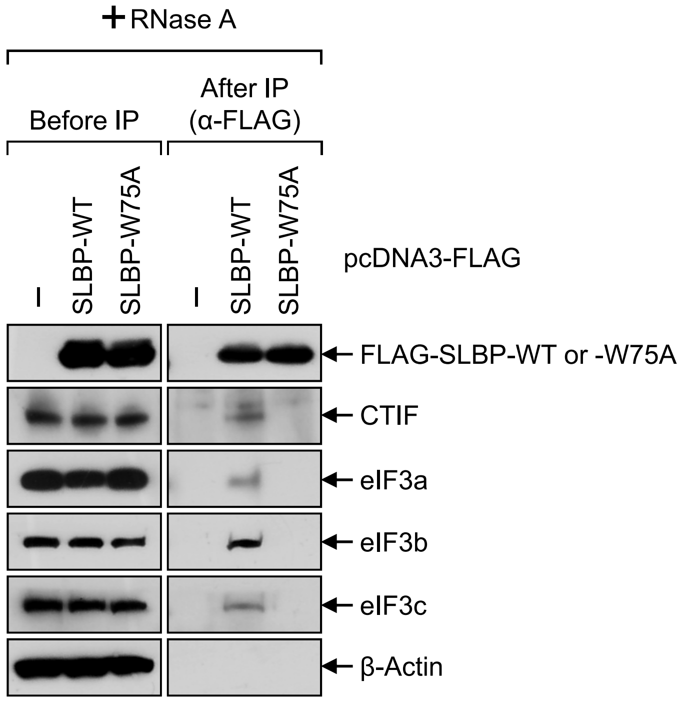
+ RNase A
Before IP
After IP (α-FLAG)
–
SLBP-WT
SLBP-W75A
–
SLBP-WT
SLBP-W75A
pcDNA3-FLAG
FLAG-SLBP-WT or -W75A
CTIF
eIF3a
eIF3b
eIF3c
β-Actin

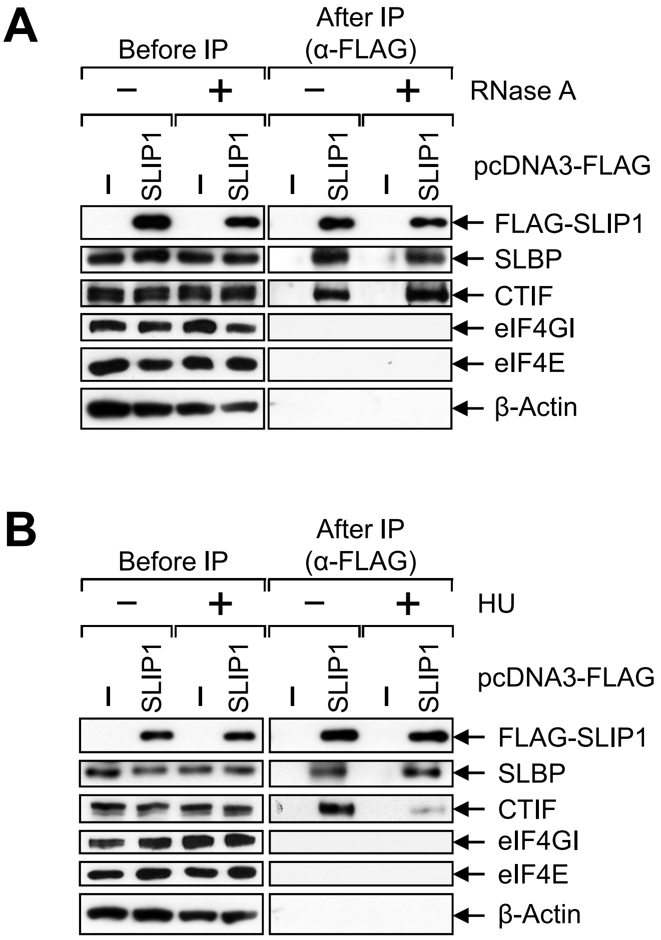
A
Before IP
After IP
(α-FLAG)
RNase A
pcDNA3-FLAG
SLIP1
FLAG-SLIP1
SLBP
CTIF
eIF4GI
eIF4E
β-Actin
B
Before IP
After IP
(α-FLAG)
HU
pcDNA3-FLAG
SLIP1
FLAG-SLIP1
SLBP
CTIF
eIF4GI
eIF4E
β-Actin

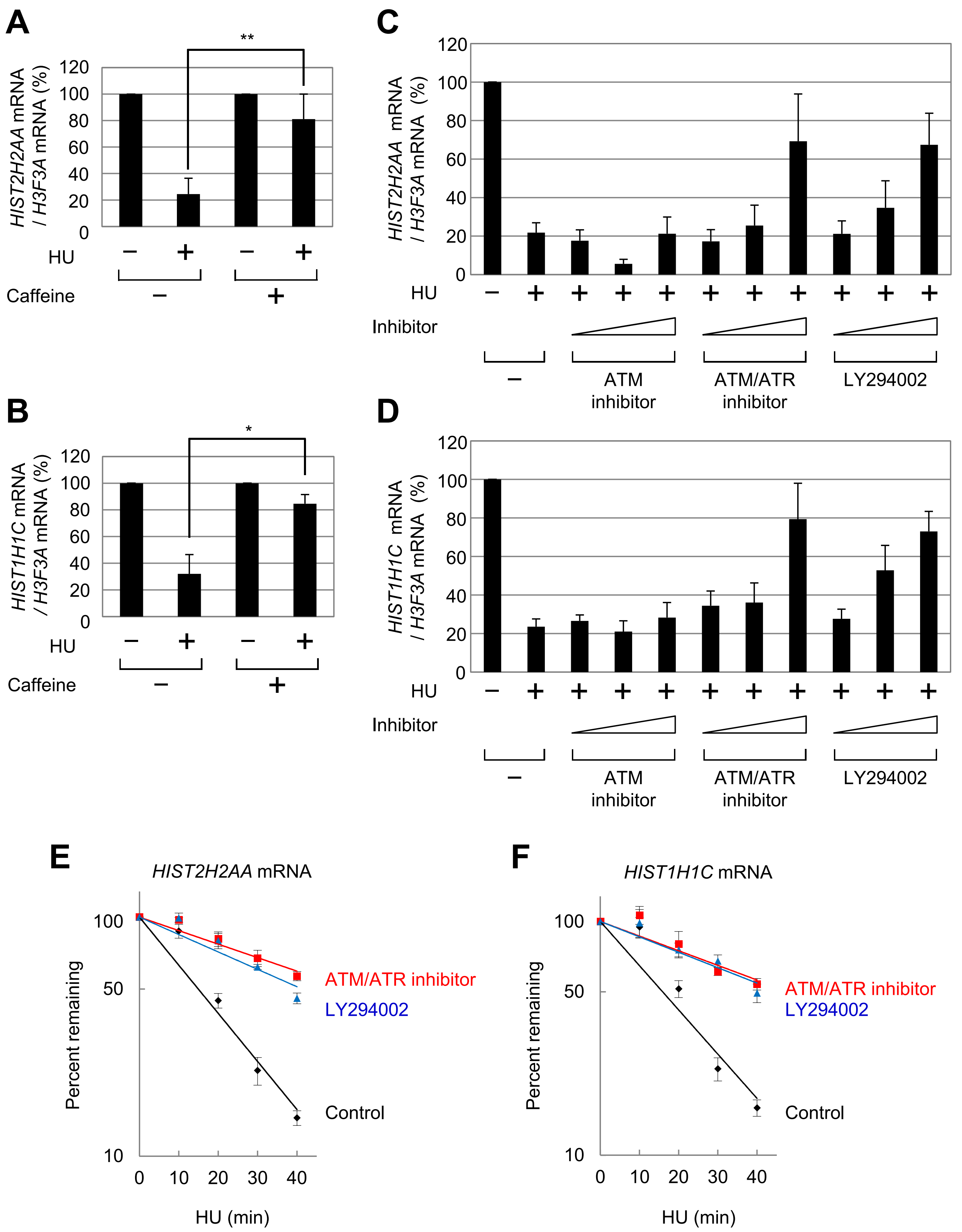
A
**
HIST2H2AA mRNA / H3F3A mRNA (%)
120
100
80
60
40
20
0
HU
−
+
−
+
Caffeine
−
+
B
*
HIST1H1C mRNA / H3F3A mRNA (%)
C
HIST2H2AA mRNA / H3F3A mRNA (%)
Inhibitor
ATM inhibitor
ATM/ATR inhibitor
LY294002
D
HIST1H1C mRNA / H3F3A mRNA (%)
E
HIST2H2AA mRNA
Percent remaining
10
50
100
0
10
20
30
40
HU (min)
ATM/ATR inhibitor
LY294002
Control
F
HIST1H1C mRNA

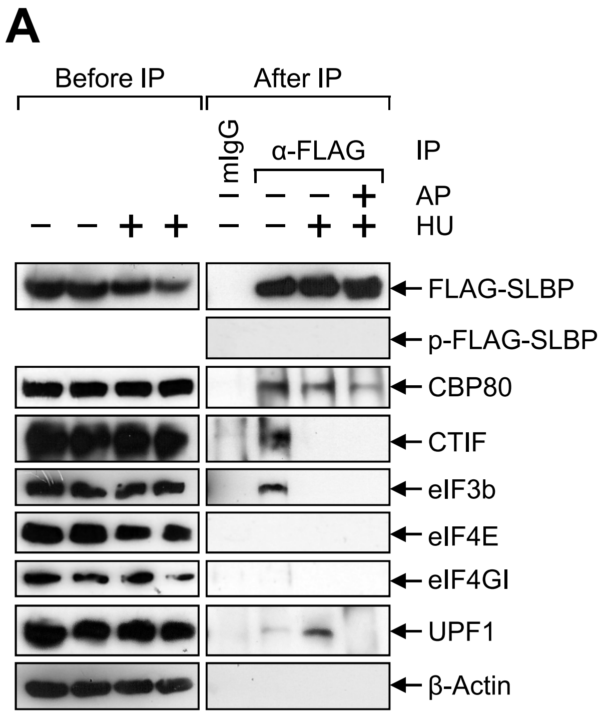
A
Before IP
After IP
mIgG
α-FLAG
IP
AP
HU
FLAG-SLBP
p-FLAG-SLBP
CBP80
CTIF
eIF3b
eIF4E
eIF4GI
UPF1
β-Actin
B
Before IP
After IP
mIgG
α-FLAG
IP
AP
HU
FLAG-CTIF
p-FLAG-CTIF
SLBP
SLIP1
CBP80
eIF4E
eIF4GI
β-Actin

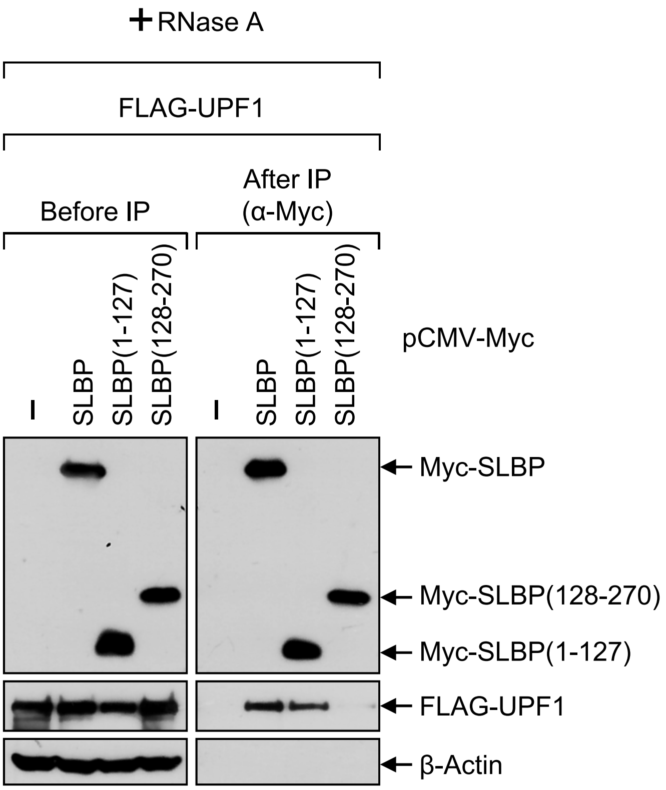
+ RNase A
FLAG-UPF1
Before IP
After IP
(α-Myc)
pCMV-Myc
-
SLBP
SLBP(1-127)
SLBP(128-270)
-
SLBP
SLBP(1-127)
SLBP(128-270)
Myc-SLBP
Myc-SLBP(128-270)
Myc-SLBP(1-127)
FLAG-UPF1
β-Actin

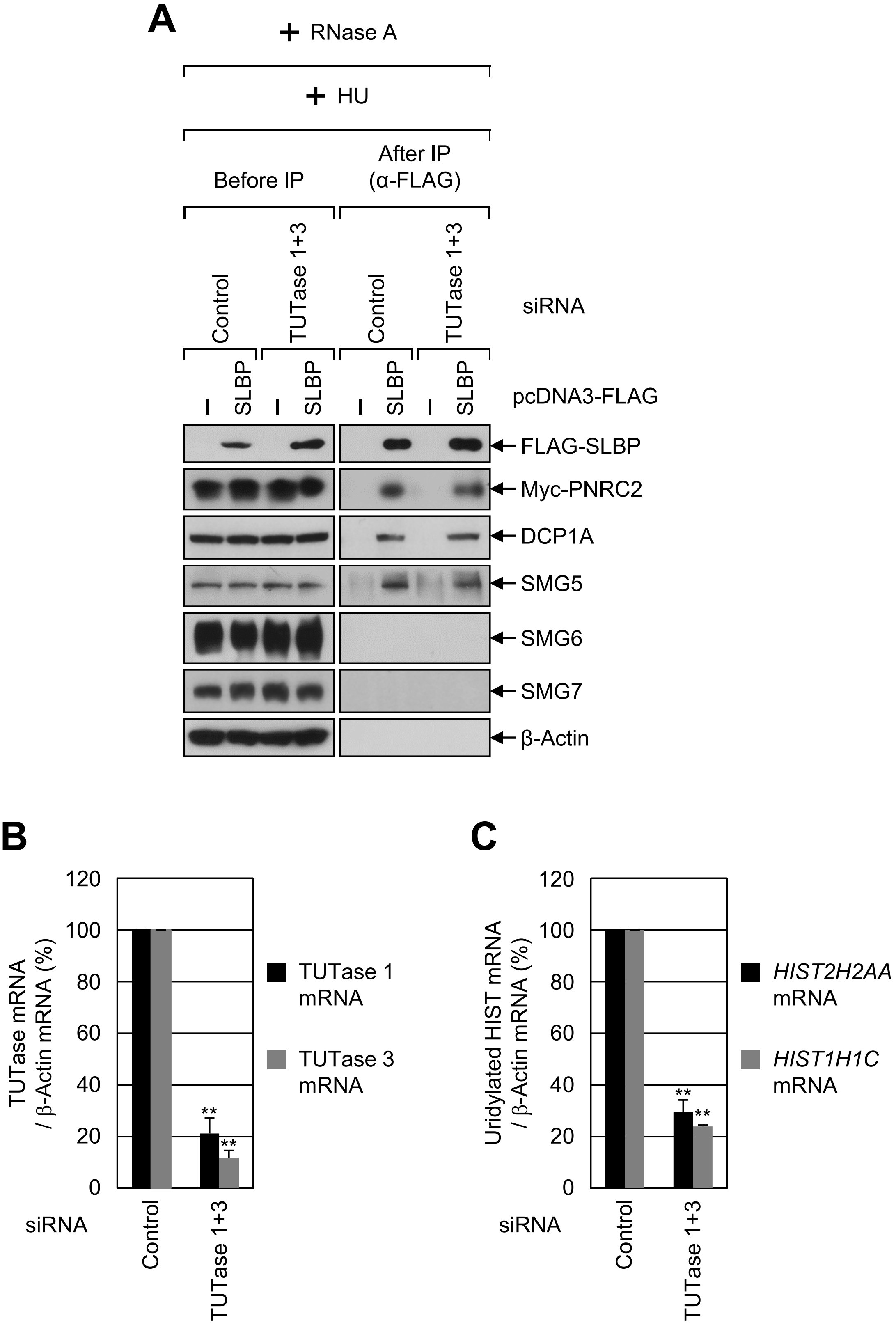
A
+ RNase A
+ HU
Before IP
After IP (α-FLAG)
Control
TUTase 1+3
siRNA
SLBP
pcDNA3-FLAG
FLAG-SLBP
Myc-PNRC2
DCP1A
SMG5
SMG6
SMG7
β-Actin
B
TUTase mRNA / β-Actin mRNA (%)
0
20
40
60
80
100
120
**
TUTase 1 mRNA
TUTase 3 mRNA
siRNA
Control
TUTase 1+3
C
Uridylated HIST mRNA / β-Actin mRNA (%)
HIST2H2AA mRNA
HIST1H1C mRNA
siRNA
Control
TUTase 1+3
